# A novel hybrid PSO based on levy flight and wavelet mutation for global optimization

**DOI:** 10.1371/journal.pone.0279572

**Published:** 2023-01-06

**Authors:** Yong Gao, Hao Zhang, Yingying Duan, Huaifeng Zhang

**Affiliations:** 1 Department of Electronic Engineering, Ocean University of China, Qingdao, China; 2 Department of Electrical and Computer Engineering, University of Victoria, Victoria, BC, Canada; Karlstad University: Karlstads Universitet, SWEDEN

## Abstract

The concise concept and good optimization performance are the advantages of particle swarm optimization algorithm (PSO), which makes it widely used in many fields. However, when solving complex multimodal optimization problems, it is easy to fall into early convergence. The rapid loss of population diversity is one of the important reasons why the PSO algorithm falls into early convergence. For this reason, this paper attempts to combine the PSO algorithm with wavelet theory and levy flight theory to propose a new hybrid algorithm called PSOLFWM. It applies the random wandering of levy flight and the mutation operation of wavelet theory to enhance the population diversity and seeking performance of the PSO to make it search more efficiently in the solution space to obtain higher quality solutions. A series of classical test functions and 19 optimization algorithms proposed in recent years are used to evaluate the optimization performance accuracy of the proposed method. The experimental results show that the proposed algorithm is superior to the comparison method in terms of convergence speed and convergence accuracy. The success of the high-dimensional function test and dynamic shift performance test further verifies that the proposed algorithm has higher search stability and anti-interference performance than the comparison algorithm. More importantly, both t-Test and Wilcoxon’s rank sum test statistical analyses were carried out. The results show that there are significant differences between the proposed algorithm and other comparison algorithms at the significance level *α* = 0.05, and the performance is better than other comparison algorithms.

## Introduction

Optimization problems are widely found in signal processing, image processing, automatic control, and many other fields. With the emergence of complex combinatorial optimization problems, traditional optimization methods suffer from the inability to complete the problem in a short period of time and are prone to the phenomenon of “combinatorial explosion“. Population intelligence is a computational technique based on the behavioral laws of biological groups, which provides ideas for solving complex distributed problems.

In recent years, many metaheuristic algorithms based on animal behavior in nature have been proposed by researchers, such as the particle swarm algorithm (PSO) [1] that simulates the foraging process of a flock of birds; the ant colony algorithm (ACO) [2] that simulates ants finding the shortest path from a food source to a nest; the gray wolf optimizer (GWO) [3] that imitates the social hierarchy and navigation mechanism of grey wolves in nature; the whale optimization algorithm (WOA) [4] that is inspired by the humpback whale bubble net foraging and the ant-lion optimizer (ALO) [5] imitating the hunting mechanism of ant lion in nature. As well, the salp swarm algorithm (SSA) [6] that is primarily inspired by the clustering behavior of salmon as they navigate and feed in the ocean; The artificial gorilla troop optimizer (AGTO) [7] inspired by the social intelligence of gorilla troops in nature; The african vulture optimization algorithm (AVAO) [8] that simulates the foraging and navigation behavior of african vultures; The bald eagle search Algorithm (BES) [9] that simulates the hunting strategy or intelligent social behavior of vultures when searching for fish; The moth flame optimizer (MFO) [10] that mimics the navigation methods of moths in nature; The sperm swarm optimizer (SSO) [11], inspired by the dynamics of sperm fertilizing egg cells, and the dragonfly algorithm (DA) [12], inspired by the static and dynamic swarming behavior of dragonflies in nature, etc. Researchers have also focused their research points to physical facts, such as reference to mathematical models based on sine and cosine functions fluctuating outward or toward the optimal solution, to emphasize the exploration of the search space at different stages of optimization by the sine and cosine optimizer (SCA) [13]; The arithmetic optimization algorithm (AOA) [14] that exploits the distributional behavior of the main arithmetic operators in mathematics (multiplication (M), division (D), subtraction (S), and addition (A)); the gravitational search algorithm (GSA) [15] based on the law of universal gravitation and mass interaction; The main inspiration comes from the three concepts of white hole, black hole and wormhole in cosmology, and their mathematical models are used to perform multi-verse optimizer (MVO) [16] for exploration, development and local search, respectively; The cooperative search algorithm (CSA) [17], inspired by the cooperative behavior of modern corporate teams and the proposed hybrid algorithm AGSA-PS [18] by combining the adaptive gravitational search algorithm (AGSA) and pattern search (PS); The hybrid optimization algorithm HSSOGSA [19], which combines the gravitational search algorithm (GSA) and sperm swarm optimization (SSO), to combine the advantages of both algorithms, etc.

As mentioned above, almost all metaheuristic algorithms mimic the processes of selecting and adapting to the environment that already exist in nature and model them as two search behaviors, exploitation and exploration. Among the known meta-heuristic algorithms, the PSO algorithm based on swarm intelligence proposed by Kennedy et al. in 1995 has been applied to many fields because of its simple structure and easy implementation. However, the particle swarm optimization algorithm has the problem of premature convergence into local optimum and slow convergence when approaching or entering the optimal region when solving high-dimensional complex problems. In order to overcome these problems, literature review found that researchers mainly focus on two improvement methods. One is to improve the optimization effect of particle swarm by improving the parameter update method in the particle swarm update formula. As in the literature [20] proposes a time-varying acceleration factor that allows the algorithm to improve by focusing on the learning of *Pbest* in the early stages of evolution and on *Gbest* in the later stages. The literature [21] accomplishes the particle position update in the form of Gaussian sampling by eliminating the velocity term of traditional PSO, which makes the algorithm structure more concise and easy to operate. To make the particle swarm more applicable to the dynamic environment, the updating method of inertia weights becomes the main improvement direction. For example, the literature [22] proposes a stochastic inertia weight update method; the literature [23] proposes a linear inertia weight for balancing the needs of global search in the early stage and local search in the later stage; and the literature [24] treats the success rate as a feedback coefficient and proposes an adaptive inertia weighting technique.

The other one is to enhance the search capability by meritively combining with other optimization algorithms to form a new hybrid algorithm. As in literature [25] combines PSO with DE algorithm and proposes a hybrid DE and PSO (DEPSO) algorithm to solve the economic scheduling problem. A hybrid algorithm of hybrid PSO and GSA is proposed in document [26], and the performance is verified and analyzed using the benchmark function. The literature [27] uses Levy flight combined into PSO to update the position equations and enhance the global search capability. In the literature [28], a new hybrid particle swarm optimization method is proposed which combines wavelet theory based variational operations to enhance the performance of exploring the solution space. Similarly, the literature [29] uses Gaussian distribution to update the PSO position formula without parameter adjustment. In recent years, there are many other ways to improve PSO search performance by combining, such as the combination of PSO and GA [30], the combination of PSO and ACO [31], the combination of PSO and ACO [32], the combination of PSO and SCA [33], the combination of PSO and SCA and levy flight [34], the combination of PSO and BFO [35], the combination of PSO and WOA [36], the combination of PSO and MFB [37] and so on.

In this paper, a PSOLFWM algorithm is proposed for the purpose of solving PSO early convergence and adapting to nonlinear complex multidimensional problems. The algorithm combines the Levy distribution and wavelet theory to update the position formula of PSO. In this method, PSO runs in the direction of the improved vector, and the random walk characteristic of Levy flight makes the search space of PSO larger. Wavelet theory is used as a mutation operator to modify decision vector, increase convergence stability and improve the quality of solution space. The proposed algorithm has been tested using well-known mathematical test functions. The results show the accuracy and robustness of the method in complex optimization problems, and it is a very effective search algorithm.

The remainder of this paper is organized as follows. Section 2 describes the preparation of PSO, wavelet theory, and Levy flight. Section 3 introduces the proposed optimization technology. Section 4 describes the experimental results and discussion of the proposed algorithm. The conclusions are given in Section 5.

## Preliminaries

In this section, we briefly introduce the basic framework of PSO, Levy flight, Wavelet Mutation, and some basic concepts.

### Particle swarm optimization

Particle swarm optimization (PSO) is a random search algorithm derived from the study of bird predation behavior. Particle swarm optimization algorithm often uses two concepts: exploration, which means that particles leave the original search track to a certain extent and search in a new direction, reflecting the ability to explore unknown areas. The other is exploitation, which means that the particles continue to search in a finer step on the original search trajectory to some extent, mainly referring to further exploration of the area searched during the exploration. All particles adjust their motion in real time according to the velocity and position [38] of Eqs 1 and 2.

$$\begin{array}{c} V_{id}(t+1)=wV_{id}(t)+c_{1}rand(P_{id}(t)-X_{id}(t))+c_{2}rand(P_{gd}(t)-X_{id}(t)) \end{array}$$ (1)

$$\begin{array}{c} X_{id}(t+1)=X_{id}(t)+V_{id}(t+1) \end{array}$$ (2)

where *X*_*id*_ and *V*_*id*_ are the position and velocity components of the *i*th particle in the *d*th dimension, respectively. *P*_*id*_ and *P*_*gd*_ are the local optimal solution of the *i*th individual particle and the global optimal solution of the particle population, respectively. *c*_1_ and *c*_2_ are cognitive acceleration factors. *w* is the inertia weight, which determines the inheritance of the current velocity of the particle,updated by the equation Eq 3.


$$\begin{array}{c} w=w_{\text{max}}-\frac{(w_{\text{max}}-w_{\text{min}})iter}{Max\_iter} \end{array}$$ (3)

Subsequently, Clerc et al. [39] expanded the search space to improve the quality of the solution by adding a constraint factor to the velocity update formula. The velocity update formula for the compression factor method is.

$$\begin{array}{c} V_{id}(t+1)=\lambda \{V_{id}(t)+c_{1}r_{1}(P_{id}(t)-X_{id}(t))+c_{2}r_{2}(P_{gd}(t)-X_{id}(t))\} \end{array}$$ (4)

$$\begin{array}{c} \lambda =\frac{2}{\left|2-\zeta -\sqrt{\zeta^{2}-4\zeta }\right|} \end{array}$$ (5)

where *λ* is the compression factor and *ζ*=*c*_1_+*c*_2_.

### Wavelet mutation (WM)

Wavelet analysis is a rapidly growing new field in applied mathematics and engineering disciplines. After years of exploration and research, the formal system of mathematics has been established with a solid theoretical foundation. Wavelet transform can carry out a multi-scale detailed analysis of functions or signals through the operation functions of scaling and translation. This solves many difficulties and problems that Fourier transform can not solve. A particle swarm optimization algorithm based on wavelet mutation is proposed [28], whose mutation function acts as a fine-tuning of the particles. Assuming that $X_{i}^{k}=(x_{i1}^{k},x_{i2}^{k},\cdots ,x_{id}^{k})$ is the *i*th particle at the *k*th iteration and $x_{i\chi }^{k}$ is the *χ*th dimension (1 ≤ *χ* ≤ *d*) of that particle, *ub* and *lb* are the upper and lower bounds of the search space, respectively, the formula is updated as follows.

$$\begin{array}{c} mut(x_{i\chi }^{k})=\{\begin{array}{c} x_{i\chi }^{k}+\sigma (ub-x_{i\chi }^{k}),\sigma >0\\ x_{i\chi }^{k}+\sigma (x_{i\chi }^{k}-lb),\sigma \leq 0 \end{array} \end{array}$$ (6)

where $mut(x_{i\chi }^{k})$ represents the value after the mutation. *σ* is the wavelet function value, and when *σ* is close to 1, the closer the variational particle value is to the maximum value *ub*. When *σ* is close to −1, the closer the variant particle value is to the lower limit *lb*. It can be seen that *σ* determines the size of the search space to some extent. Morlet wavelet function is selected here, and its calculation formula is as follows:

$$\begin{array}{c} \sigma =\frac{1}{\sqrt{a}}e^{\left(-{\left(\frac{\varphi }{a}\right)}^{2}/2\right)}\text{cos}\left(\frac{5\varphi }{a}\right) \end{array}$$ (7)

where *φ* takes values in the range of pseudo-random numbers in the interval [−2.5*a*, 2.5*a*]. The *a* is called the scale parameter and is calculated as follows.

$$\begin{array}{c} a=e^{-\text{ln}\;g\times {\left(1-\frac{iter}{\text{max}\_iter}\right)}^{\zeta_{wm}}} \end{array}$$ (8)

where *ζ*_*wm*_ is the monotonically increasing shape parameter and *g* is the upper limit of the parameter *a*. The mutation operation using wavelet theory has good convergence ability on the basis of the stability of the improved algorithm. In this paper, we set *g* = 10000 and *sigma* = 5.

### Levy flight

Levy flight is a random walk whose step length follows the Levy distribution. The Levy distribution differs from the normal and Cauchy distributions in that it is a heavy-tailed distribution with a higher probability of being in the same position. For the same conditions, the Levy flight under the Levy distribution has a much larger search area than the Brownian motion under the uniform distribution. The Levy distribution can be expressed by a mathematical formula as follows.

$$\begin{array}{c} L\left(s,\gamma ,\mu \right)=\left\{\begin{array}{ll} \sqrt{\frac{\gamma }{2\pi }}\;\text{exp}\left(-\frac{\gamma }{2\left(s-\mu \right)}\right)\frac{1}{{\left(s-\mu \right)}^{\frac{3}{2}}}, & 0<\mu <\infty \\ 0, & otherwise \end{array}\right\} \end{array}$$ (9)

where *μ* and *s* are the transmission parameters and samples, respectively. The step length of Levy flight can be calculated as follows according to Mantegna’s algorithm.

$$\begin{array}{c} s=\frac{u}{{\left|v\right|}^{\frac{1}{\beta }}} \end{array}$$ (10)

where the variables *u* and *v* are following a normal distribution, as shown in the following equation, and *β* is a fixed parameter.

$$\begin{array}{c} u∼N(0,\sigma_{u}^{2}),\;v∼N(0,\sigma_{v}^{2}) \end{array}$$ (11)

where *σ*_*v*_ = 1, and the variable *σ*_*u*_ is updated by the following equation.

$$\begin{array}{c} \sigma_{u}={\left(\frac{\Gamma (1+\beta )\;\text{sin}\;(\frac{\pi \beta }{2})}{\Gamma (\frac{1+\beta }{2})\;\beta 2^{\left(\frac{\beta -1}{2}\right)}}\right)}^{\frac{1}{\beta }} \end{array}$$ (12)

In this paper, we set *scale* = 0.01, so the step size of the search space is *stepsize* = 0.01 * *s*.

## Hybrid PSO based on levy flight and wavelet mutation

In meta heuristic algorithm, particle swarm optimization algorithm is a kind of population optimization algorithm with fast calculation speed, few parameters and simple implementation. However, the two defects of easy premature convergence and falling into local minima limit its wide application to a certain extent. In view of this, many researchers have made many improvements to the particle swarm algorithm. Since the use of Levy flight allows for a more efficient search in the search space. In the literature [27, 40, 41], the researchers achieved an improvement of the PSO algorithm by combining its velocity and position equations with Levy flight.

As shown in Section 2.1, the compression factor PSO expands the search space to some extent. In order to further increase the quality of the solution space, we try to combine the compression factor PSO and levy flight. The speed and position update formulas are as follows.

$$\begin{array}{c} V_{id}(t+1)=\lambda \{wLevy(X_{id}(t),\text{dim})+\varphi_{1}rand(P_{id}(t)-X_{id}(t))+\varphi_{2}rand(P_{gd}(t)-X_{id}(t))\} \end{array}$$ (13)

$$\begin{array}{c} X_{id}(t+1)=V_{id}(t+1) \end{array}$$ (14)

In the above equation, *Levy* (*X*_*id*_ (*t*), dim) is the expression for the combination of Levy flight and PSO, which is calculated as shown in the literature [27].

$$\begin{array}{c} Levy(X_{id}(t),\text{dim})=X_{id}(t)+\text{step}⊕\text{randn}(\text{size}(X_{id}(t))) \end{array}$$ (15)

$$\begin{array}{c} \text{step}=stepsize⊕X_{id}(t) \end{array}$$ (16)

where *dim* is the dimension of the optimization search problem, ⊕ is the element multiplication, and *w* is calculated based on Eq 3.

Considering that, as described in Section 2.2, wavelet mutation can improve the algorithm’s stability and have good convergence ability. For example, in the literature [28, 42], researchers use the mutation function of wavelet change to fine-tune the particle swarm by combining the position update equation of particle swarm with wavelet change. Here, to generate more solutions of different schemes in the whole design space, improve the quality of solution space and increase the fine-tuning stability of the algorithm, Levy flight is considered to be added to wavelet mutation. The update formula is as follows.

$$\begin{array}{c} X_{id}(t+1)=\{\begin{array}{c} Levy(X_{id}(t),\text{dim})+\sigma (ub-X_{id}(t)),\sigma >0\\ Levy(X_{id}(t),\text{dim})+\sigma (X_{id}(t)-lb),\sigma \leq 0 \end{array} \end{array}$$ (17)

In the above equation, *σ* is calculated based on the Eq 7.

Therefore, we give the pseudo-code of the proposed algorithm PSOLFWM as shown in Algorithm 1. Where, *SearchAgents*_*no* indicates population size, *Max*_*iteration* is the maximum number of iterations, *dim* is the dimension of the optimization problem, *ub*, *lb* is the upper and lower limits of the particle search space, *Vmax*,*Vmin*,*wmax*,*wmin* are the upper and lower limits of the flight speed and the upper and lower limits of the inertia weight during particle search, *φ*_1_, *φ*_2_ is the scaling factor. After the initialization of the algorithm is completed, the inertia weight and scaling factor used in the algorithm are updated by using the formulas Eqs 3 and 5. We use the method of setting *trial* in the literature [27] for reference, and judge the update method of the selected location through the size range of *trial*. A penalty mechanism is added to increase diversity by reinitializing the population when *trial* is greater than 10*limit and starting to update the position according to Eq 13.

Algorithm 1 Pseudo code of the PSOLFWM algorithm.

**Initailize**:

1: Initailize parameters: *SearchAgents*_*no*, *Max*_*iteration*, *dim*, *ub*, *lb*

2: ,*Vmax*, *Vmin*, *wmax*, *wmin*, *φ*_1_, *φ*_2_, *trial*, *limit*

3: Initailize populations:*Positions*, *velocity*

4: Initailize individual&population optimum:*Pbest*, *Gbest*

**Main Loop**:

5: **while** *iter* < *Max*_*iteration* **do**

6:  **for** *j* = 1 : *SearchAgents*_*no* **do**

7:   Update *w* with Eq 3

8:   Update *λ* with Eq 5

9:   **if** (*trial* < *limit*) **then**

10:    Update *Positions*(*j*, :), *velocity*(*j*, :) with Eq 13, Eq 14

11:   **else if** (*limit* ≤ *trial* < 10*limit) **then**

12:    **if** *σ* > 0 **then**

13:     Update *Positions*(*j*, :) with Eq 17

14:    **else**

15:     Update *Positions*(*j*, :) with Eq 15

16:    **end if**

17:   **else**

18:    ReInitial *velocity*(*j*, :) and *Positions*(*j*, :)

19:    ReUpdate *velocity*(*j*, :) and *Positions*(*j*, :) with Eq 13, Eq 2

20:   **end if**

21:   Boundary processing

22:   **if** (*fobj*(*Positions*(*j*, :)) < *Pbest*(*j*)) **then**

23:    *trial* = 0

24:    *p*(*j*, :) = *Positions*(*j*, :)

25:    *Pbest*(*j*) = *fobj*(*Positions*(*j*, :))

26:   **else**

27:    *trial* = *trial* + 1

28:   **end if**

29:   Update global optima(*Gbest*)

30:  **end for**

31:  Record the *Gbest* solution

32:  *iter* = *iter* + 1

33: **end while**

34: Output the global best (*Gbest*) solution

With the repeated operation of the algorithm, the particle updates the velocity and position continuously. The individual optimum *Pbest* of the group of particles is selected by performing a comparison of the fitness value *fobj*(*Positions*(*j*, :)) with the individual optimum *Pbest*(*i*). Subsequently, by comparing the current individual optimum *Pbest* with the global optimum *Gbest*, the global optimum *Gbest* is selected and assigned to the global optimum *Gbest*. The program stops when the maximum number of iterations is reached or when the set optimal fitness value is obtained, the global optimum is output and the fitness value convergence curve is plotted as required.

## Experimental results and discussion

In this section, the performance of the proposed PSOLFWM algorithm will be evaluated. For performance comparison, two types of algorithms are reproduced and used. They are improved particle swarm family optimization algorithms based on particle swarm algorithm, including particle swarm algorithm (PSO), standard particle swarm algorithm (SPSO), mutation operator particle swarm algorithm (HPSOM), wavelet mutation particle swarm algorithm (HPSOWM), bone backbone particle swarm algorithm (BBPSO), Levy flying particle swarm algorithm (PSOLF), Levy flying sine cosine particle swarm algorithm (PSOSCALF) and gray wolf particle swarm algorithm (PSOGWO). The other is other meta-heuristic optimization algorithms, including the gray wolf optimization algorithm (GWO), the differential evolution algorithm (DE), the sine cosine optimization algorithm (SCA), the whale optimization algorithm (WOA), the ant-lion optimization algorithm (ALO), the salp swarm algorithm (SSA), the dragonfly algorithm (DA), the moth flame optimizer (MFO), the bald eagle search optimization algorithm (BES), the cooperative search algorithm (CSA), and the sperm swarm optimization algorithm (SSO). Aiming at the stochastic nature of the optimization algorithms, their performance is evaluated using a set of benchmark functions with different characteristics. All algorithms were performed in Windows 10 OS using Inter Core i5, 3.3GHz, 16GB RAM.

### Parameter settings

Table 1 reveals the empirically obtained control parameters affecting the performance of the PSOLFWM method. The control parameters of some of the particle swarm family algorithm and other meta-inspired optimization algorithms used as comparison algorithms are shown in Table 2.

**Table 1:** PSOLFWM parameter setting.

Parameters	Value	Parameters	Value
Vmax	10	beta	1.5
Vmin	-10	scale	0.01
phi1	2.5	g	10000
phi2	2.5	sigma	5
wmax	1.2	SearchAgents_no	50
wmin	0.1	Max_iteration	100
RunNo	30		

**Table 2:** Comparison algorithm control parameters.

Algorithm	Parameters	Value	Parameters	Value	Parameters	Value
CommonParas	Vmax	10	phi1	2.5	wmax	1.2
	Vmin	-10	phi2	2.5	wmin	0.1
PSO [43]	w	0.9	c1	1.5	c2	1.5
	c1max	2.5	c2max	2.5	wmax	0.9
PSOSCALF [34]	c1min	0.5	c2min	0.5	wmin	0.4
	k	10	limit	10		
DE [44]	beta_max	0.8	beta_min	0.2	pCR	0.2

### Benchmark functions

In this paper, 21 benchmark functions in three categories are given in S1 Appendix for evaluating the proposed PSOLFWM algorithm. The first is a unimodal function with a single optimal solution (F1-F7), the second is a multimodal function with multiple local optimal solutions (F8-F13), and the third is a fixed dimensional multimodal function (F14-F21). The mathematical expression (Column 1), dimension (Column 2), search space range and initialization range (Column 3) and global optimal solution (Column 4) of the function are given in S1 Appendix.

### Comparison of algorithm accuracy

Solution accuracy is an important index to measure the algorithm. In this subsection, the performance metrics of the proposed algorithm in terms of convergence accuracy are measured using 21 benchmark test functions. To better verify the superiority of the proposed algorithm, the optimization performance of the proposed algorithm is compared with eight particle swarm optimization algorithms and eleven meta-heuristic optimization algorithms under the same test function.

#### Comparison of particle swarm family algorithms

Since the particle swarm optimization algorithm was proposed, it has been improved by many researchers and finally formed the particle swarm family optimization algorithm. This subsection completes the performance comparison with the proposed optimization algorithm by reproducing eight particle swarm family optimization algorithms. They are PSO [38], SPSO [45], HPSOM [46], HPSOWM [28], BBPSO [21], PSOLF [41], PSOGWO [47] and PSOSCALF [34].

In this paper, we carry out the optimization test for 21 tested benchmark functions with a population size of 50, a single maximum iteration of 200, and 30 runs. S2–S4 Appendices gives the indicator data collected during the optimization process for each algorithm to find the best value, including the mean, standard variance value, median value, optimal value, worst value, average running time, and algorithm classification. The classification basis of the algorithm in this paper is to sort according to the minimum order of mean value and standard variance. We bolded all individuals with the best value in the comparison algorithm, and those with an algorithm grading of 1. The shortest average running time was italicized and bolded and underlined.

**Fig 1:**
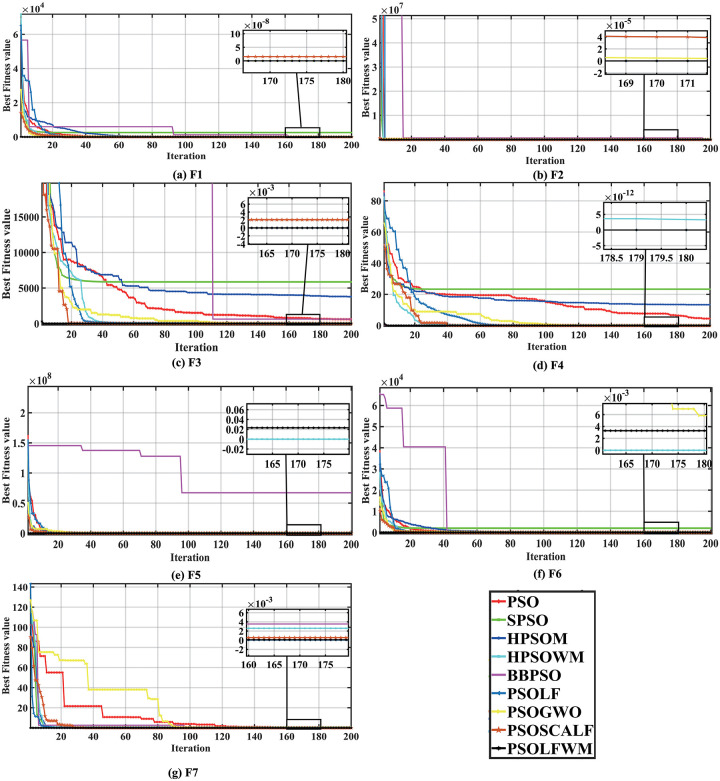
Convergence curve of unimodal functions compared with the PSO family algorithms. (a) F1, (b) F2, (c) F3, (d) F4, (e) F5, (f) F6, and (g) F7.

As shown in S2 Appendix, for the F1-F7 unimodal benchmark test functions, the proposed PSOLFWM algorithm is able to find the optimal values quickly. Among them, the five functions F1, F2, F3, F4, and F7 find the optimal value in the average value, standard deviation, median value, optimal value, and worst value. However, F5 has the best mean, standard deviation, median, and minimum worst value in addition to the optimal values. The best standard deviation shows that it has better search stability than HPSOWM. Therefore, the PSOLFWM algorithm ranks first in the hierarchical ranking, which is more robust than other algorithms. For function F6, PSOLFWM search ability is a bit weaker compared to HPSOWM, and the algorithm ranking is 2nd place. From the average running time of 30 iterations, it can be concluded that PSO has the fastest running time, BBPSO takes the longest time to run, and the proposed algorithm PSOLFWM has an average running time in the middle, which is within the acceptable range. From the convergence curves of the fitness values in Fig 1, we can see that the PSOLFWM has the best convergence speed and accuracy when performing the optimization search of these six functions. The search process for F6 is very smooth. In the early stage, the global search effect is good, but in the later stage, it falls into a local minimum value very close to 0. The final optimal value is slightly worse compared to the algorithm HPSOWM.

**Fig 2:**
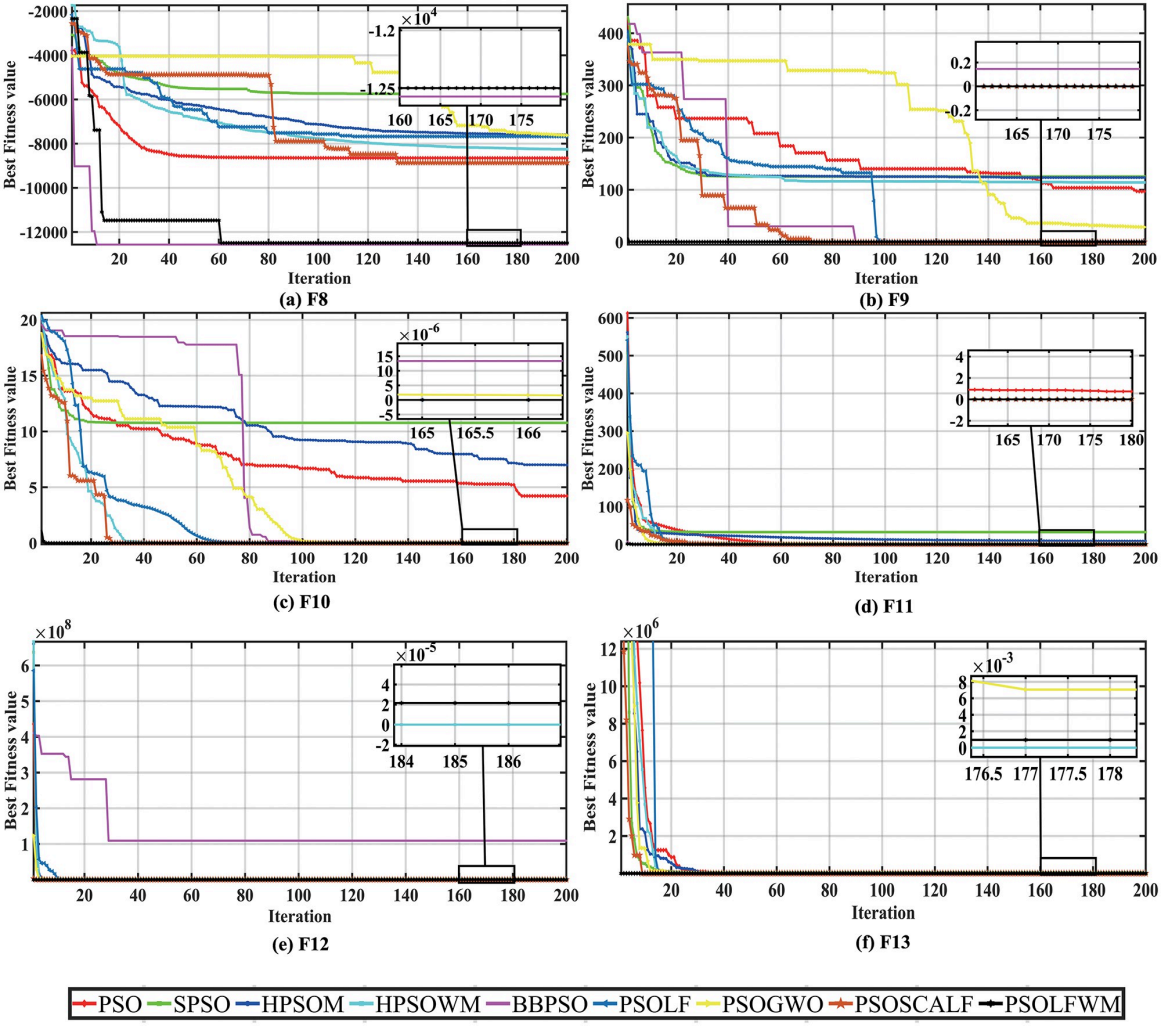
Convergence curve of multimodal functions compared with the PSO family algorithms. (a) F8, (b) F9, (c) F10, (d) F11, (e) F12, and (f) F13.

In the multimodal function test of F8-F13, although the multimodal function has multiple locally optimal solutions, the proposed algorithm can still complete the problem optimization. From the S3 Appendix, we can see that the BBPSO algorithm performs well in optimizing F8 and F11 functions, ranking first. The HPSOWM algorithm performs well in finding the optimal F12,F13 function and ranks first. The PSOLF algorithm and the proposed PSOLFWM algorithm are perfect in the five performance metrics when optimizing multimodal functions such as F9, F10 and F11, and rank first. The proposed algorithm achieves a suboptimal value in the optimization of F8, F12 and F13 functions, and the algorithm ranks second. As seen from the convergence curve Fig 2, the proposed algorithm is slightly insufficient in the optimization process of F8, F12 and F13 functions. However, it is still in the second-ranking position in terms of standard deviation and other indicators, and the search process is relatively stable. Therefore, when optimizing the multimodal function, the convergence speed and convergence accuracy of the proposed algorithm are better than other algorithms to a certain extent, and the average running time is in the average level.

**Fig 3:**
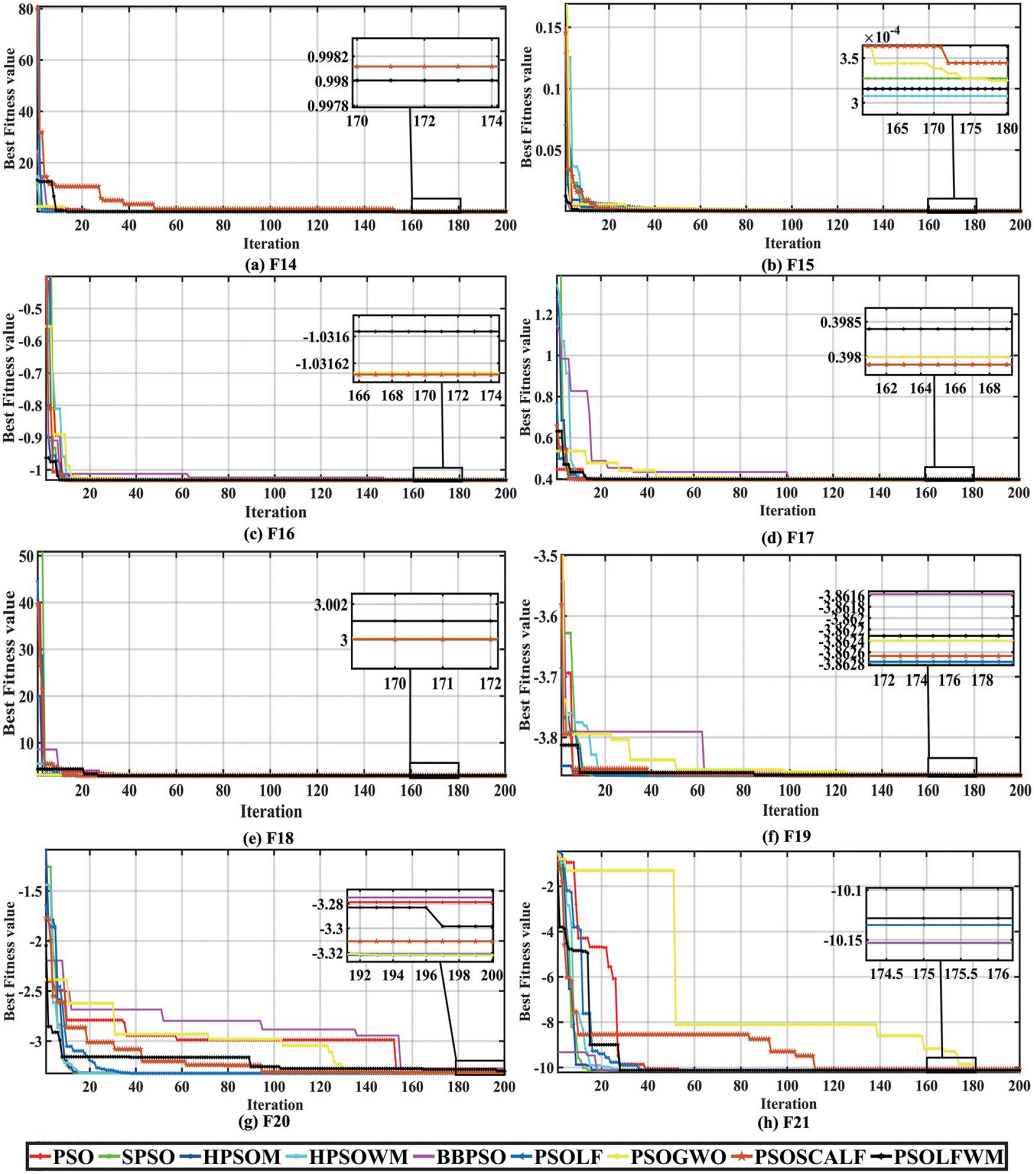
Convergence curve of others functions compared with the PSO family algorithms. (a) F14, (b) F15, (c) F16, (d) F17, (e) F18, (f) F19, (g) F20, and (h) F21.

As shown in Fig 3, for the F14-F21 test function, the proposed algorithm can complete the global optimization within 200 iterations. However, in the algorithm grading with standard deviation as the criterion performance generally only F14 is ranked first, F15, F21 is ranked second. Nevertheless, the standard deviation of the proposed algorithm performs generally in the F16-F20 test function. By carefully observing the S4 Appendix, it is found that the proposed algorithm obtains the same optimal value as other algorithms, and has faster convergence speed and convergence accuracy. For the average running time, the PSO algorithm is the fastest to complete 200 iterations, which also explains, to some extent, the low complexity of the algorithm.

#### Comparison with other meta-heuristics

After completing the comparison with the particle swarm optimization algorithm, to further verify the effectiveness of the algorithm, this paper also lists and compares eight meta-heuristic algorithms, which are GWO [3], DE [48], SCA [13], WOA [4], ALO [5], SSA [6], DA [12], MFO [10], BES [9], CSA [17], and SSO [49]. These algorithms are different from particle swarm optimization and are also popular swarm intelligence algorithms among researchers in recent years.

The comparison method is the same as that of particle swarm optimization. For all algorithms, set the maximum number of iterations to 200, the number of single runs to 30, and the population size to 50. The initial values of the search space are randomly generated from the initial population. S5–S7 Appendices gives the performance index results of the proposed algorithm and eleven meta-heuristic algorithms when optimizing 21 test benchmark functions. In the same way, as in S2–S4 Appendices, the best solution in the table is shown in bold, and the average running time of the algorithm is shown in italics with bold underlines.

**Fig 4:**
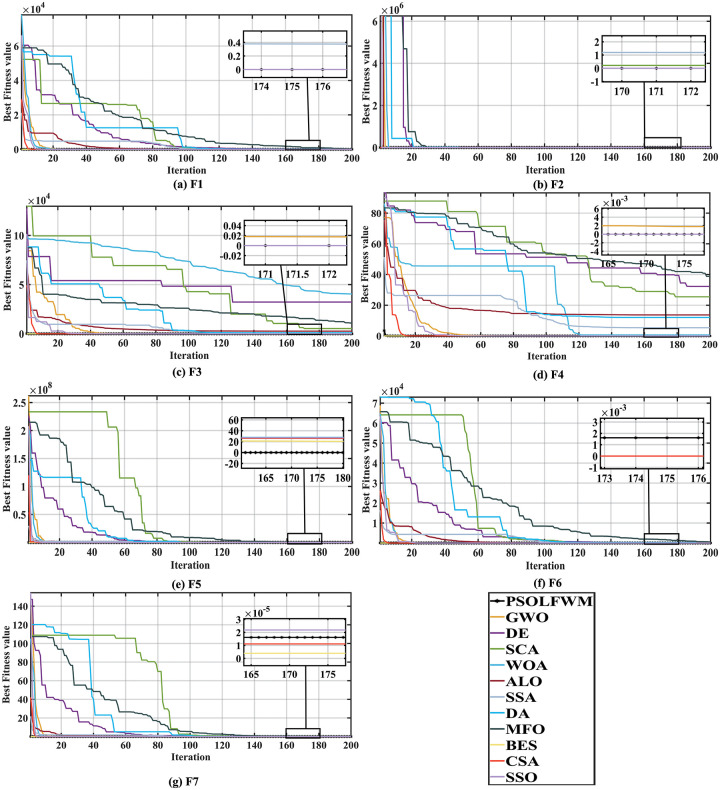
Convergence curve of unimodal functions compared with the meta-heuristic algorithms. (a) F1, (b) F2, (c) F3, (d) F4, (e) F5, (f) F6, and (g) F7.

S5 Appendix shows the optimization results of the above optimization algorithm for the unimodal functions F1-F7. From the table, we can understand that the proposed algorithm performs exceptionally well in terms of mean, standard variance, median, best and worst values for F1, F2, F3, F4, F5 of the unimodal function, and the algorithm ranks first in the classification. The BES algorithm ranked first under the condition that the standard deviation is the grading criterion of the algorithm when the functions F1,F2,F3,F4,F6, and F7 are searched for superiority. However, comparing the average running time of the proposed algorithm PSOLFWM and BES, it can be found that the proposed algorithm can obtain the optimal value in the shortest time and converge earlier. Although the ability of the proposed PSOLFWM to obtain the optimal value when optimizing the F6,F7 functions is poor compared to the BES and CSA algorithms. Yet, for F7, the proposed algorithm is weaker than BES and SSA algorithms by an order of magnitude of 1e-5, and convergence to zero is acceptable. From Fig 4, it can be seen that the convergence curves of the proposed algorithm in F1-F7 compared with other meta-algorithms converge quickly and efficiently to the function extremum 0.

**Fig 5:**
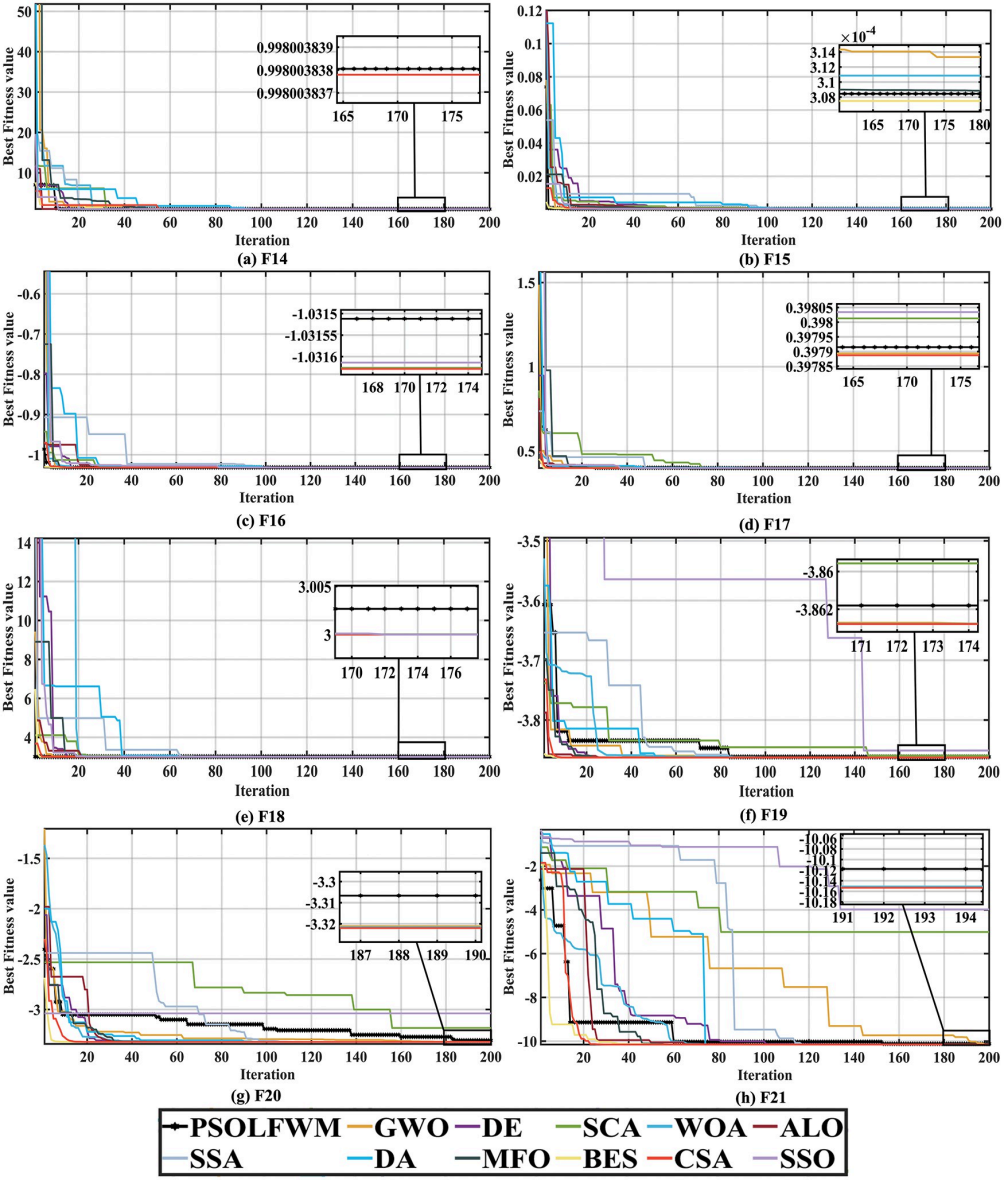
Convergence curve of multimodal functions compared with the meta-heuristic algorithms. (a) F14, (b) F15, (c) F16, (d) F17, (e) F18, (f) F19, (g) F20, and (h) F21.

Similarly, for multimodal functions, the proposed algorithm can still maintain the optimal solution compared with other meta heuristic algorithms. It can be seen from S6 Appendix that the proposed algorithm shows excellent optimization ability in F9-F13 (except F12), ranking first. For function F8, the algorithm WOA converges to the global optimum at the earliest and is ranked first, with the PSOLFWM algorithm ranked second. PSOLFWM, BES, CSA, and SSO achieved the same global optimal values in all metrics in F9, F11, and ranked first in the hierarchical ranking. However, in terms of average running time, the proposed algorithm PSOLFWM takes less time and converges to the global optimum faster. When performing global search for F12, the proposed algorithm PSOLFWM converges to the global optimum value of 0 by an order of magnitude of 1e-4 within 200 iterations, although it is weaker than the BES,CSA algorithm ranked in the the third position. The algorithm WOA takes the shortest time in the optimization search process from F8-F13, indicating the simplicity of the algorithm structure and also the tendency to fall into premature convergence. According to Fig 5, the proposed algorithm exhibits the best convergence speed and accuracy for both F8-F13.

**Fig 6:**
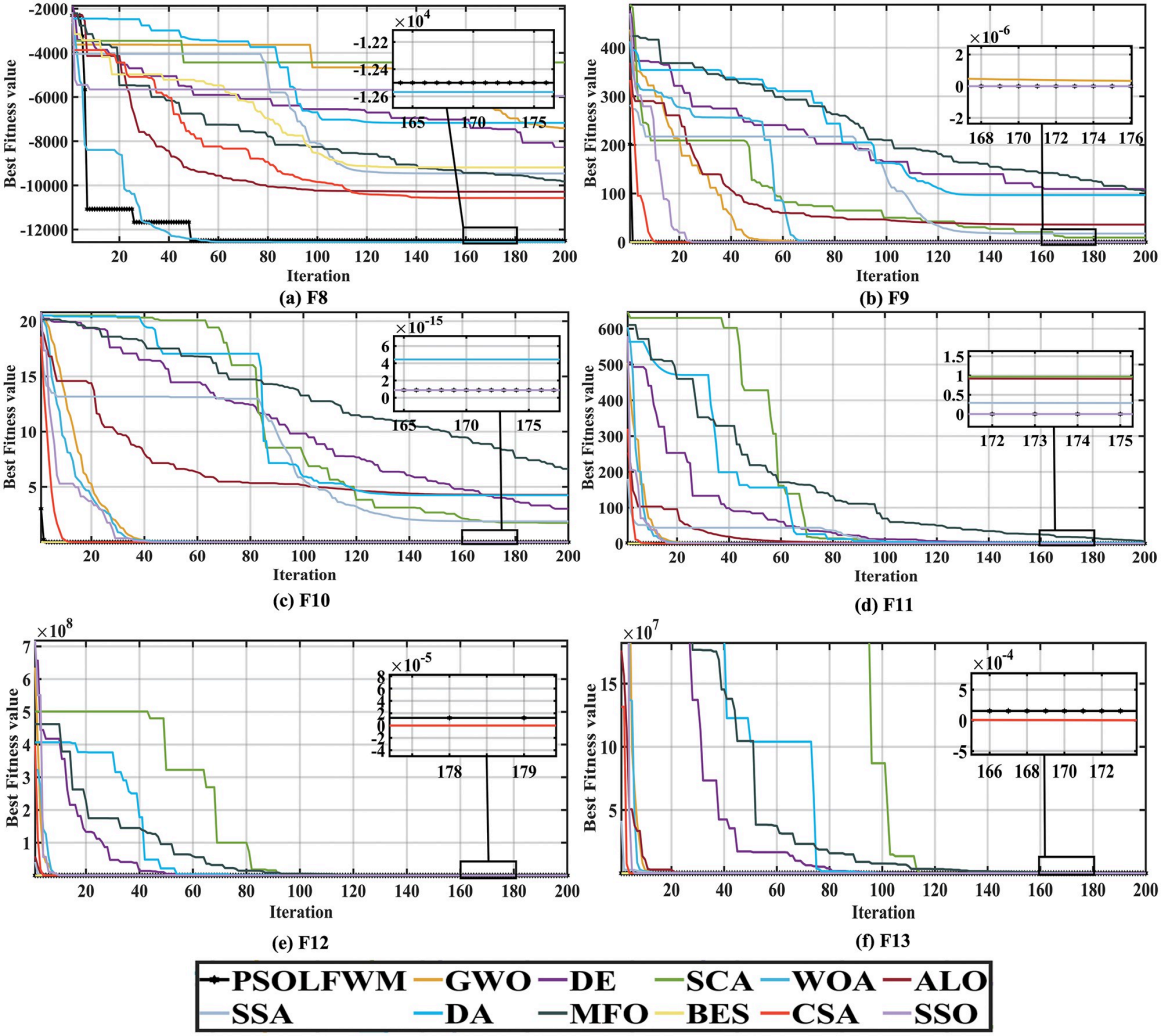
Convergence curve of others functions compared with the meta-heuristic algorithms. (a) F8, (b) F9, (c) F10, (d) F11, (e) F12, and (f) F13.

F14-F21 convergence curves for the test function are given in Fig 6. As can be seen from the figure, all optimization algorithms complete the search for the global optimum within a finite number of iterations. It has been found that the proposed algorithm performs better for the test functions F14,F15, and F21 by further analyzing the results of the search values given in S7 Appendix. Sorted by the global optimal value of the standard deviation, the DE algorithm is ranked first in the search for the optimal F14,F16,F17,F18, and F20 functions, the MFO is ranked first in the search for the optimal F17, and F19, the BES is ranked first in the search for the optimal F17, and the CSA is ranked first in the search for the optimal F17, and F21. Although the proposed algorithm ranks lower in F16, F17, F18, F19, and F20, it has little difference from the optimal global solution, and all obtain the global optimal or suboptimal values. And the ranking backward is related to the choice of our ranking rules and does not precisely represent the degree of superiority of the algorithm. As shown in Fig 6, the proposed algorithm searches steadily throughout the process. For the average running time, we can see from the table that the WOA algorithm takes the least time, i.e., it completes 200 search iterations the fastest.

### Statistical analysis tests

t-Test [43] is a common statistical method used to distinguish between the superiority and inferiority of two algorithms. If the result *t* value is positive, it means that the optimization performance of algorithm *x* is better than that of algorithm *y*, and conversely, the optimization performance of algorithm *y* is better than that of the algorithm.

$$\begin{array}{c} t=\frac{\bar{x}-\bar{y}}{\sqrt{\left(\frac{s_{x}^{2}}{n})+(\frac{s_{y}^{2}}{m}\right)}} \end{array}$$ (18)

In Eq 18, $\bar{x}$ and $\bar{y}$ denote the sample means of algorithm *x* and algorithm *y*, respectively, *s*_*x*_ and *s*_*y*_ are the sample standard variances of algorithm *x* and algorithm *y*, and *n* and *m* denote the sample sizes. It can be seen from Tables 3, 4 that the proposed PSOLFWM algorithm, whether compared with the particle swarm optimization algorithm or meta-heuristic algorithm, most of the t-values are greater than zero, which can show good optimization performance. The NaN term indicates that both sides of the comparison algorithm can reach the optimal value within a finite number of iterations, but it does not apply to t-Test.

**Table 3:** Comparison of PSOLFWM with PSO families algorithms using t-Test.

Functions	PSO [38]	SPSO [45]	HPSOM [46]	HPSOWM [28]	BBPSO [21]	PSOLF [41]	PSOSCALF [34]	PSOGWO [47]
F1	**1.6488E+01**	**1.8504E+01**	**2.0764E+01**	**1.6950E+00**	**4.5633E+00**	**4.8423E+00**	**2.2971E+00**	**6.9683E+00**
F2	**2.1697E+01**	**2.2996E+01**	**2.4070E+01**	**1.0029E+00**	**4.7627E+00**	**6.1614E+00**	**2.6500E+00**	**7.4962E+00**
F3	**1.5939E+01**	**1.8195E+01**	**1.4838E+01**	**6.1753E+00**	**1.9865E+01**	**2.5819E+00**	**2.8508E+00**	**5.4027E+00**
F4	**1.5665E+01**	**4.2407E+01**	**3.0752E+01**	**1.2902E+00**	**1.7984E+00**	**3.4697E+00**	**4.8350E+00**	**8.2209E+00**
F5	**1.1581E+01**	**1.0248E+01**	**7.7921E+00**	**1.5781E+01**	**3.1704E+01**	**2.9972E+02**	**3.1467E+02**	**1.4328E+02**
F6	**1.5321E+01**	**1.8200E+01**	**2.2453E+01**	-6.8381E+00	**3.6493E+00**	**3.3131E+01**	**2.0479E+01**	**1.0064E+00**
F7	**1.1261E+01**	**1.0743E+01**	**1.2016E+01**	**6.5211E+00**	**4.9414E+00**	**2.5150E-01**	**6.2029E+00**	**1.3113E+01**
F8	**1.4345E+01**	**2.4341E+01**	**1.7558E+01**	**1.4613E+01**	**-6.3294E+00**	**1.7740E+01**	**1.5082E+01**	**1.7395E+01**
F9	**4.5873E+01**	**3.5241E+01**	**5.2457E+01**	**5.1027E+01**	**4.6559E+00**	NaN	**4.0941E+00**	**1.6292E+01**
F10	**3.9566E+01**	**6.6082E+01**	**3.3650E+01**	**1.8676E+00**	**5.3439E+00**	NaN	**6.0986E+00**	**7.8993E+00**
F11	**1.5146E+02**	**2.2858E+01**	**1.9253E+01**	**2.1315E+00**	NaN	NaN	**4.7724E+00**	**6.2235E+00**
F12	**1.1812E+01**	**3.7097E+00**	**4.0770E+00**	-8.9249E+00	**2.6143E+01**	**1.4227E+01**	**1.5409E+01**	**3.4318E+00**
F13	**2.2334E+00**	**4.8690E+00**	**3.8871E+00**	-4.7105E+00	**3.6475E+01**	**5.6893E+02**	**1.3834E+01**	**6.8110E+00**
F14	**1.0000E+00**	**8.1059E+00**	**4.9970E+00**	**5.4518E+00**	**1.1201E+00**	**3.5349E+00**	**1.9521E+00**	**1.7970E+00**
F15	**6.8777E+00**	**1.9267E+00**	**3.5675E+00**	**1.5665E+00**	**4.0570E+01**	**3.1233E+00**	**5.4318E+00**	**2.6792E+00**
F16	-6.6418E+00	-6.6453E+00	-6.6289E+00	-6.6453E+00	**4.0930E+00**	-6.6423E+00	-5.9722E+00	-6.5655E+00
F17	-4.8945E+00	-4.8954E+00	-4.8917E+00	-4.8954E+00	**6.3169E+00**	-4.8324E+00	-2.8063E+00	-4.8723E+00
F18	-6.4253E+00	-6.4303E+00	-6.4287E+00	-6.4303E+00	**4.1595E+00**	-6.4274E+00	-6.2658E+00	-6.4300E+00
F19	-3.4594E+00	-6.2007E+00	-5.8063E+00	-6.2012E+00	**2.1434E+00**	-5.3564E+00	-2.2466E+00	-6.1796E+00
F20	**3.1378E+00**	-2.2258E+00	-1.0751E+00	-1.4046E+00	**7.9559E+00**	-4.8138E+00	-4.9540E-01	-2.6751E+00
F21	**2.5366E+00**	**7.7940E+00**	**3.8377E+00**	**4.5568E+00**	-6.8228E+00	**2.2835E+00**	**3.7193E+00**	**6.0450E-01**

**Table 4:** Comparison of PSOLFWM with other meta-heuristic algorithms using t-Test.

Functions	GWO [3]	DE [48]	SCA [13]	WOA [4]	ALO [5]	SSA [6]	DA [12]	MFO [10]	BES [9]	CSA [17]	SSO [49]
F1	**7.2373E+00**	**2.4460E+01**	**5.2340E+00**	**2.2918E+00**	**4.0827E+00**	**8.6979E+00**	**6.0579E+00**	**3.6847E+00**	NaN	**1.4243E+00**	**1.1891E+00**
F2	**1.0556E+01**	**4.7819E+01**	**8.4938E+00**	**1.7152E+00**	**7.2908E+00**	**1.3097E+01**	**1.4337E+01**	**1.1974E+01**	**6.5535E+04**	**5.7612E+00**	**1.2407E+00**
F3	**4.6561E+00**	**4.7077E+01**	**9.8953E+00**	**2.1801E+01**	**1.0995E+01**	**1.3056E+01**	**8.7959E+00**	**1.7710E+01**	NaN	**1.4590E+00**	**1.2060E+00**
F4	**1.0921E+01**	**5.6488E+01**	**3.0914E+01**	**7.2560E+00**	**2.8724E+01**	**1.6165E+01**	**2.0733E+01**	**3.5825E+01**	**6.5535E+04**	**3.6840E+00**	**1.0990E+00**
F5	**1.8709E+02**	**1.6204E+01**	**4.0956E+00**	**3.3671E+02**	**4.3071E+00**	**4.8916E+00**	**5.3632E+00**	**6.8440E+00**	**1.4496E+02**	**4.6284E+02**	**3.0525E+02**
F6	**9.8842E+00**	**2.1770E+01**	**7.1782E+00**	**1.3231E+01**	**4.3356E+00**	**7.8211E+00**	**8.5123E+00**	**3.5934E+00**	-7.0267E+00	-7.0265E+00	**1.1695E+02**
F7	**7.3184E+00**	**2.2562E+01**	**5.2612E+00**	**4.4718E+00**	**1.3686E+01**	**1.5154E+01**	**8.3971E+00**	**5.1661E+00**	-5.6182E+00	-5.1512E+00	-4.2898E+00
F8	**1.6130E+01**	**1.2801E+01**	**2.7138E+01**	**1.1997E+00**	**1.5746E+01**	**1.0960E+01**	**1.7006E+01**	**8.0877E+00**	**1.5570E+01**	**4.7113E+00**	**1.9976E+01**
F9	**5.1557E+00**	**6.3697E+01**	**1.0910E+01**	**1.3605E+00**	**1.4664E+01**	**1.6537E+01**	**2.3608E+01**	**2.5875E+01**	NaN	NaN	NaN
F10	**1.0067E+01**	**7.4375E+01**	**1.0914E+01**	**8.8773E+00**	**2.2039E+01**	**2.2856E+01**	**2.0338E+01**	**1.4946E+01**	NaN	NaN	**3.8079E+00**
F11	**3.1779E+00**	**1.1416E+02**	**5.4336E+00**	**1.0000E+00**	**8.5094E+00**	**2.6500E+01**	**8.2439E+00**	**3.9392E+00**	NaN	NaN	NaN
F12	**9.4788E+00**	**3.0238E+01**	**2.2975E+00**	**1.0232E+01**	**1.3261E+01**	**1.5707E+01**	**1.1037E+00**	**1.7656E+00**	-7.4365E+00	-7.4361E+00	**5.4048E+01**
F13	**1.4472E+01**	**2.0681E+01**	**2.5012E+00**	**1.1248E+01**	**1.9402E+00**	**1.1851E+01**	**2.5656E+00**	**4.7220E+00**	**3.3245E+01**	**3.5638E+00**	**1.8536E+02**
F14	**3.5874E+00**	-1.1029E+00	**3.6698E+00**	**3.3703E+00**	**3.4834E+00**	**2.9725E+00**	**5.1828E+00**	**3.0700E+00**	**2.9814E+00**	**3.9396E+00**	**3.9707E+00**
F15	**2.2259E+00**	**1.4159E+01**	**1.0101E+01**	**4.5329E+00**	**1.9242E+00**	**2.0369E+00**	**3.0103E+00**	**7.4243E+00**	-8.9610E-01	**8.4449E+00**	**1.7947E+00**
F16	-5.2390E+00	-5.2394E+00	-4.8818E+00	-5.2394E+00	-5.2394E+00	-5.2394E+00	-5.2394E+00	-5.2394E+00	-5.2394E+00	-5.2346E+00	-3.6182E+00
F17	-4.5856E+00	-4.5963E+00	**2.8307E+00**	-4.5270E+00	-4.5963E+00	-4.5963E+00	-4.5963E+00	-4.5963E+00	-4.5963E+00	-4.5963E+00	**4.7844E+00**
F18	-4.9806E+00	-4.9898E+00	-4.9545E+00	-4.9830E+00	-4.9898E+00	-4.9898E+00	-4.9898E+00	-4.9898E+00	-4.9898E+00	-4.9898E+00	-4.9866E+00
F19	-6.7442E+00	-9.1643E+00	**1.6080E+00**	-7.0710E-01	-9.1643E+00	-9.1635E+00	-6.6795E+00	-9.1643E+00	-9.1643E+00	-9.1643E+00	**5.2461E+00**
F20	-4.1811E+00	-8.5130E+00	**4.8197E+00**	-2.4638E+00	-3.6237E+00	-1.7947E+00	-2.2297E+00	-2.4935E+00	-5.0735E+00	-3.3242E+00	**1.1510E+01**
F21	**1.7847E+00**	-7.7050E-01	**2.3165E+01**	**3.3057E+00**	**9.3750E+00**	**4.2338E+00**	**5.4320E+00**	**5.8259E+00**	**3.8570E+00**	-6.1652E+00	**5.6844E+01**

To further determine whether the results of the PSOLFWM optimization algorithm obtained in the previous sections are statistically significant compared to the results of other comparison optimization algorithms, the Wilcoxon’s rank sum test is supplemented [50]. For this purpose, we performed a nonparametric test on the optimization result data for 30 times at the significance level *α* = 0.05. S8 Appendix reports the p, h, and z values of the Wilcoxon’s rank sum test results of PSOLFWM compared with other optimization algorithms. Similar to the t-test, the NaN entries in the table indicate that both optimization algorithms are not suitable for Wilcoxon’s rank sum test. By studying and analyzing the results in the table, it was found that when testing F9,F10, and F11 in the multimodal function, the NaN term appeared in the statistical test results, which means that the above algorithms all searched for the optimal value during the running, but the statistical test was not available. From the p-values in S8 Appendix, we know that for unimodal functions F1-F7, the PSOLFWM outperforms the other comparison algorithms (except for the PSOLF algorithm testing F7). For the multimodal functions F8-F13, the performance of PSOLFWM outperforms other comparison algorithms (except the WOA algorithm). It also means that there is a significant difference between the performance of PSOLFWM and other comparative optimization algorithms. For F14, F20, and F21 in the fixed-dimensional multimodal test function, the proposed method in this paper does not show any significant performance improvement compared with other comparative algorithms. As for all tested functions F1-F21, the performance of PSOLFWM is significantly better than that of PSO, DE and SSO algorithms. Therefore, the statistical test analysis shows that the results of the proposed algorithm in this paper are significantly better than the comparative optimization algorithm cited in this paper.

### Comparison of high-dimensional function tests

In the face of large and complex problems, the traditional intelligent optimization algorithm often needs to traverse the whole search space, resulting in the combination explosion phenomenon, and can not complete the task search in the specified time. For many engineering optimization problems, such as the optimization and design of the complex controller, its search space is ample, and there are strict requirements for calculation speed and convergence. Therefore, the optimization ability of high-dimensional functions must be considered in the design of an optimization algorithm. In order to verify the optimization performance of the proposed algorithm for high-dimensional functions, six typical test benchmark functions of three types, F1, F3, F7, F9, F12, and F15, with 200, 500, 800, 1000, 2000, and 3000 dimensions, are selected in this subsection. The test performance metrics are the average number of iterations, the average convergence time, and the average convergence rate within the convergence threshold of the optimized value obtained by designing to satisfy Eqs 19 and 20.

Property 1.

$$\begin{array}{c} |F^{*}-F_{best}|\leq V_{accept} \end{array}$$ (19)

Property 2.

$$\begin{array}{c} Ratio=\frac{N_{s}}{N} \end{array}$$ (20)

where F* is the global optimum, *F*_*best*_ is the optimum obtained in a single iteration, *V*_*accept*_ is the designed convergence threshold, and *N*_*s*_ is the number of successful convergences in *N* runs. This paper is designed to automatically exit the current iteration when the algorithm reaches the convergence threshold. Set the maximum number of iterations of the algorithm to 500 steps and the number of test runs of the algorithm to 50. The given high-dimensional functions and convergence thresholds are shown in Table 5.

**Table 5:** Test functions and convergence thresholds.

Functions	F1	F3	F7	F9	F12	F15
Vaccept	1.00E-05	1.00E-05	1.00E-05	1.00E-05	1.00E-07	1.00E-05

**Fig 7:**
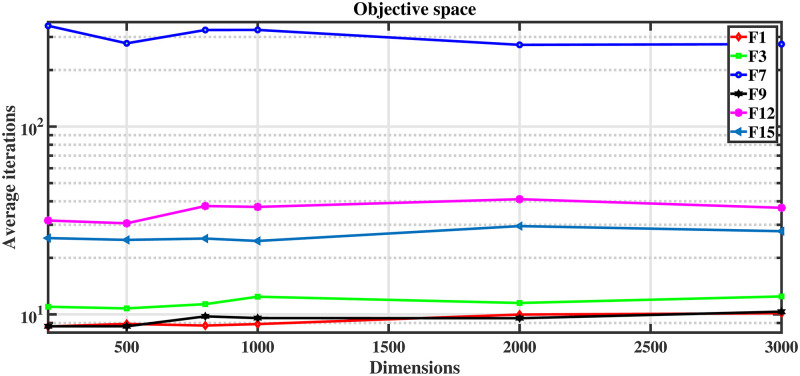
Relationship curve between iteration times and function dimension.

As can be seen from the data in Table 6, the six high-dimensional test functions we selected all converge to an acceptable range, while their number of iterations is not large. As seen in Fig 7, the average number of iterations of the proposed algorithm PSOLFWM for seeking optimization does not occur significantly with the increase of the dimensionality. For the ultra-high-dimensional function with dimension dim = 3000, the optimization search can also be completed with almost the same number of iterations, which indicates that the proposed algorithm has obvious advantages in the optimization of high-dimensional functions with high unit search efficiency. Also, as the number of dimensions increases, the optimization runtime increases accordingly. This is because the increase in the number of dimensions makes the computational complexity increase, resulting in a corresponding increase in the search time, but the time cost is still acceptable. The above experiments have verified that PSOLFWM has a strong search capability for high-dimensional systems.

**Table 6:** High-dimensional function test results (50 tests).

	Functions	Dim = 200	Dim = 500	Dim = 800	Dim = 1000	Dim = 2000	Dim = 3000	FuncMinVal
F1	Average_iter	8.6200E+00	8.9000E+00	8.7200E+00	8.8800E+00	9.9800E+00	1.0140E+01	0.00E+00
	Average_RunTime	2.0560E-02	4.6753E-02	4.1239E-02	5.0344E-02	1.1293E-01	1.8393E-01	
	BestVal	1.5890E-15	4.2572E-16	5.5905E-16	9.5100E-16	6.7880E-17	5.6965E-19	
	Radios	100%	100%	100%	100%	100%	100%	
F3	Average_iter	1.0980E+01	1.0760E+01	1.1340E+01	1.2420E+01	1.1500E+01	1.2460E+01	0.00E+00
	Average_RunTime	1.0380E-01	3.4305E-01	7.5293E-01	1.1739E+00	3.7091E+00	9.0462E+00	
	BestVal	4.8122E-23	3.6972E-19	8.5470E-18	6.7233E-18	1.7850E-19	2.0260E-22	
	Radios	100%	100%	100%	100%	100%	100%	
F7	Average_iter	3.4400E+02	2.7744E+02	3.2668E+02	3.2702E+02	2.7232E+02	2.7462E+02	0.00E+00
	Average_RunTime	1.3394E+00	1.7973E+00	2.1449E+00	2.5101E+00	3.9902E+00	6.0906E+00	
	BestVal	1.4550E-05	6.4511E-06	3.5767E-05	6.4342E-06	6.8841E-06	4.4124E-06	
	Radios	100%	100%	100%	100%	100%	100%	
F9	Average_iter	8.6400E+00	8.6400E+00	9.7600E+00	9.5600E+00	9.5400E+00	1.0340E+01	0.00E+00
	Average_RunTime	2.2530E-02	5.2363E-02	5.4129E-02	6.6483E-02	1.0990E-01	1.7801E-01	
	BestVal	0.0000E+00	0.0000E+00	0.0000E+00	0.0000E+00	0.0000E+00	0.0000E+00	
	Radios	100%	100%	100%	100%	100%	100%	
F12	Average_iter	3.1600E+01	3.0540E+01	3.7740E+01	3.7340E+01	4.1040E+01	3.6940E+01	0.00E+00
	Average_RunTime	1.9446E-01	4.7983E-01	3.7085E-01	4.2926E-01	8.8355E-01	1.2642E+00	
	BestVal	1.0506E-04	1.9297E-04	1.3171E-04	1.3964E-04	1.6331E-04	1.2760E-04	
	Radios	100%	100%	100%	100%	100%	100%	
F15	Average_iter	2.5480E+01	2.4920E+01	2.5340E+01	2.4600E+01	2.9520E+01	2.7720E+01	3.0000E-04
	Average_RunTime	5.5261E-02	1.2127E-01	1.0324E-01	1.2099E-01	2.7708E-01	4.3218E-01	
	BestVal	3.1035E-04	3.1155E-04	3.1135E-04	3.1124E-04	3.1601E-04	3.1813E-04	
	Radios	100%	100%	100%	100%	100%	100%	

To further demonstrate the excellent high-dimensional function optimization performance of the PSOLFWM algorithm, three optimization algorithms, PSO, PSOLF, and HPSOWM, are selected as comparison algorithms to test their optimization performance in the cases of dimension 500, 1500, and 3000. The test functions still use the above three classical test functions: F1, F3, F7, F9, F12 and F15. Table 7 gives the optimization performance indexes of four optimization algorithms under high-dimensional test functions, in which the classification of algorithms is based on the optimal value of standard deviation. From the table, it can be concluded that PSO, PSOLF, HPSOWM and PSOLFWM can obtain the optimal value when the four functions of F1, F3, F7 and F9 are tested in high dimension. Among them, the PSOLFWM algorithm ranks first in classification and does not change with the increase of function dimension. In the high-dimensional test of F12 and F15 functions, it is found that the optimization performance of PSOLF algorithm decreases with the increase of dimension, while PSOLFWM can still quickly obtain the global optimal value, and the algorithm is graded as the first. The above experiments have verified that the PSOLFWM has a strong search capability for high-dimensional systems.

**Table 7:** The comparison test results of PSOLFWM and PSO, PSOLF, HPSOWM algorithms for high-dimensional functions (50 tests).

Function Name	SPI	Dim = 500				Dim = 1500				Dim = 3000			
		PSO [38]	PSOLF [41]	HPSOWM [28]	PSOLFWM	PSO	PSOLF	HPSOWM	PSOLFWM	PSO	PSOLF	HPSOWM	PSOLFWM
F1	Average	6.3743E+04	1.0731E-04	2.6326E-262	0.0000E+00	6.0586E+05	4.0879E+02	1.4863E-261	0.0000E+00	1.6285E+06	3.5918E+03	6.4323E-261	0.0000E+00
	StandDP	4.4211E+03	3.7416E-04	0.0000E+00	0.0000E+00	5.0986E+04	7.9923E+02	0.0000E+00	0.0000E+00	1.6184E+05	5.7162E+03	0.0000E+00	0.0000E+00
	BestVal	5.4481E+04	1.7607E-43	2.5315E-265	0.0000E+00	4.9239E+05	3.0065E-20	7.9517E-265	0.0000E+00	1.2775E+06	4.7514E-05	1.7482E-264	0.0000E+00
	Rank	4	3	1	1	4	3	1	1	4	3	1	1
F3	Average	1.1840E+06	1.3774E+06	5.0933E-260	0.0000E+00	1.0800E+07	2.8500E+07	8.4700E-259	0.0000E+00	4.5300E+07	1.1000E+08	8.0900E-248	0.0000E+00
	StandDP	2.3509E+05	4.2162E+05	0.0000E+00	0.0000E+00	2.3200E+06	9.4700E+06	0.0000E+00	0.0000E+00	1.3000E+07	2.8500E+07	0.0000E+00	0.0000E+00
	BestVal	6.7360E+05	1.2504E+05	2.6947E-266	0.0000E+00	7.2200E+06	1.4300E+07	1.9600E-263	0.0000E+00	2.6000E+07	6.0400E+07	1.2100E-262	0.0000E+00
	Rank	3	4	1	1	3	4	1	1	3	4	1	1
F7	Average	3.0352E+03	3.0100E-02	6.3643E-04	2.0912E-04	3.8867E+04	2.3870E-01	4.8302E-04	2.9147E-04	1.6382E+05	3.0710E-01	5.8344E-04	1.9934E-04
	StandDP	6.5078E+02	2.2600E-02	5.9563E-04	1.6638E-04	7.2962E+03	2.1540E-01	4.4989E-04	3.0805E-04	2.8287E+04	2.3560E-01	3.9055E-04	1.8036E-04
	BestVal	1.6267E+03	8.6872E-04	2.7917E-05	8.0773E-06	2.1542E+04	9.6702E-04	1.4457E-05	1.2365E-06	1.1902E+05	3.4000E-03	3.3801E-05	4.6403E-06
	Rank	4	3	2	1	4	3	2	1	4	3	2	1
F9	Average	4.4300E+03	3.9700E+03	0.0000E+00	0.0000E+00	1.4800E+04	1.4800E+04	0.0000E+00	0.0000E+00	3.0900E+04	2.8300E+04	0.0000E+00	0.0000E+00
	StandDP	1.1512E+02	1.6400E+03	0.0000E+00	0.0000E+00	2.2551E+02	3.0600E+03	0.0000E+00	0.0000E+00	3.6714E+02	9.3400E+03	0.0000E+00	0.0000E+00
	BestVal	4.0600E+03	0.0000E+00	0.0000E+00	0.0000E+00	1.4400E+04	1.0749E+00	0.0000E+00	0.0000E+00	2.9900E+04	0.0000E+00	0.0000E+00	0.0000E+00
	Rank	3	4	1	1	3	4	1	1	3	4	1	1
F12	Average	4.7225E+07	4.4709E-12	8.9890E-01	9.6649E-05	8.4009E+08	1.6361E-04	1.0562E+00	9.1482E-05	2.3469E+09	2.5470E-01	1.1121E+00	8.8745E-05
	StandDP	1.7724E+07	2.8632E-11	4.6900E-02	1.8557E-05	3.0836E+08	1.0000E-03	2.2000E-02	1.7372E-05	6.9890E+08	1.8012E+00	9.3000E-03	1.6405E-05
	BestVal	2.1009E+07	9.4233E-34	8.0690E-01	5.5268E-05	3.1555E+08	1.1339E-23	1.0025E+00	6.3222E-05	9.6508E+08	1.6112E-23	1.0941E+00	5.3913E-05
	Rank	4	1	3	2	4	2	3	1	4	3	2	1
F15	Average	5.5244E-04	7.2742E-04	5.1207E-04	3.3345E-04	5.2018E-04	1.1000E-03	5.5403E-04	3.3978E-04	5.2131E-04	1.2000E-03	4.9583E-04	3.3694E-04
	StandDP	2.2547E-04	2.6719E-04	3.7698E-04	1.6733E-05	2.3777E-04	1.1000E-03	4.0728E-04	2.7447E-05	2.3527E-04	1.9000E-03	3.5256E-04	2.8100E-05
	BestVal	3.0756E-04	3.3089E-04	3.0753E-04	3.1183E-04	3.0752E-04	3.5117E-04	3.0754E-04	3.0951E-04	3.0755E-04	3.0766E-04	3.0750E-04	3.0868E-04
	Rank	2	3	4	1	2	4	3	1	2	4	3	1

### Comparison of dynamic shift performance tests

In order to avoid the situation where the standard test function obtains the same covariate for the global optimal point, the shift function is chosen to test the dynamic optimization performance of the algorithm. The shift function is constructed according to the formula in the literature [51], where *F*(*x*) is the newly synthesized function, *f*(*x*) is the original function, *O*_old_ is the global optimum of the original function, and *O*_new_ is the global optimum of the newly synthesized function. The following six dynamic functions are selected for experimentation in this subsection, and their function expressions are shown in Table 8. Set the maximum number of iterations for a single time to 200 and run independently for 30 times.

**Table 8:** Shift test functions.

Functions_shift	[*Xmin*, *Xmax*]	Z	fmin
$F_{1}\left(x\right)=\sum_{i=1}^{n}x_{i}^{2}-450$	[-100, 100]	*Z* = *X* − *o*	-450
$F_{3}\left(x\right)=\sum_{i=1}^{n}{\left(\sum_{j-1}^{i}x_{j}\right)}^{2}-450$	[-100, 100]	*Z* = *X* − *o*	-450
$F_{7}\left(x\right)=\sum_{i=1}^{n}ix_{i}^{4}+random\left[0,1\right)-450$	[-1.28, 1.28]	*Z* = *X* − *o*	-450
$F_{9}\left(x\right)=\sum_{i=1}^{n}[x_{i}^{2}-10\;\text{cos}(2\pi x_{i})+10]-180$	[-5.12, 5.12]	*Z* = *X* − *o*	-180
$F_{10}\left(x\right)=-20\;\text{exp}(-0.2\sqrt{\frac{1}{n}\sum_{i=1}^{n}x_{i}^{2}}))-\text{exp}(\frac{1}{2}\sum_{i=1}^{2}\text{cos}\;(2\pi x_{i}))+20+e-140$	[-32, 32]	*Z* = *X* − *o*	-140
$F_{11}\left(x\right)=\frac{1}{4000}\sum_{i=1}^{n}x_{i}^{2}-\prod_{i=1}^{n}\text{cos}(\frac{x_{i}}{\sqrt{i}})+1-180$	[-600, 600]	*Z* = *X* − *o*	-180

**Fig 8:**
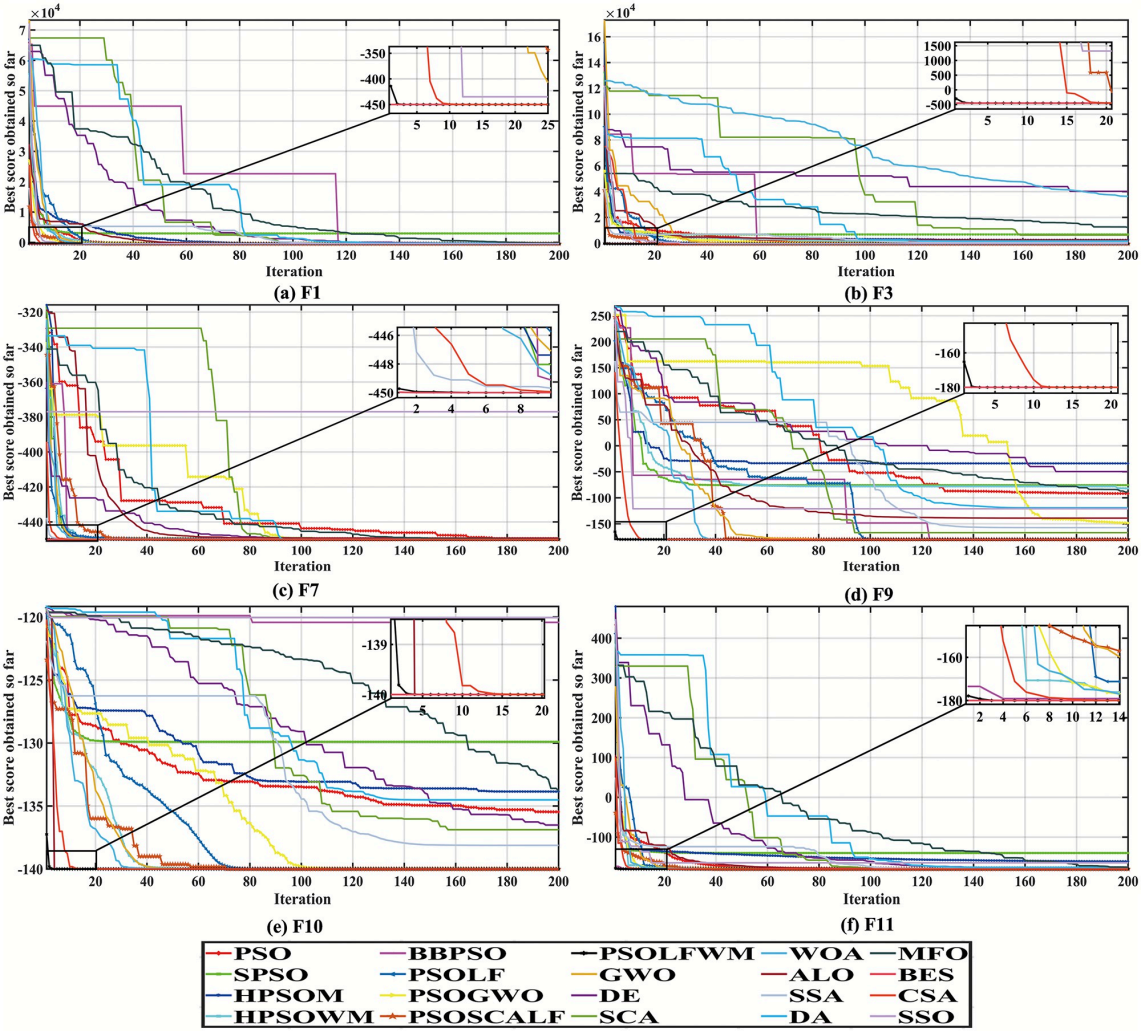
Convergence curve of shift function. (a) F1, (b) F3, (c) F7, (d) F9, (e) F10, and (f) F11.

It can be seen from Fig 8 that the proposed algorithm has excellent performance in solving shift dynamic function problem. As it is seen, the proposed algorithm has a faster convergence speed compared to the other 19 optimization algorithms.

Table 9 shows the performance results of this dynamic shift test. It can be seen from the table that the proposed algorithm is not affected by dynamic shift, and the global optimal solution is obtained within the iteration steps, which has strong anti-interference performance. Observing the test results of t-Test, it is found that the PSOLFWM shows better dynamic optimization ability than other optimization algorithms when the test functions are F1, F3, F7, F9, F10 and F11. As mentioned above, the NaN term means that t-Test does not apply to the two optimization algorithms compared. Compared with other algorithms, the proposed algorithm has good dynamic performance due to the adjustment of Levy flight distribution and wavelet function to particle swarm optimization algorithm, and dynamically balances the global and local search ability. Therefore, the proposed algorithm can better track the changes in environment and also has good stability, which is suitable for solving complex nonlinear dynamic problems.

**Table 9:** Comparison results of dynamic shift functions.

Function Name	SPI	PSO [38]	SPSO [45]	HPSOM [46]	HPSOWM [28]	BBPSO [21]	PSOLF [41]	PSOSCALF [34]	PSOGWO [47]	PSOLFWM	GWO [3]
F1	Average	-4.2914E+02	5.7797E+03	-4.3802E+02	-4.5000E+02	7.5762E+03	-4.5000E+02	-4.5000E+02	-4.5000E+02	-4.5000E+02	-4.5000E+02
	StandDP	6.2349E+00	1.7646E+03	3.5089E+00	1.8283E-14	1.3705E+04	0.0000E+00	5.7469E-05	1.1117E-11	0.0000E+00	9.5598E-11
	BestVal	-4.3699E+02	2.9601E+03	-4.4414E+02	-4.5000E+02	-4.4208E+02	-4.5000E+02	-4.5000E+02	-4.5000E+02	-4.5000E+02	-4.5000E+02
	t-Value	1.8324E+01	1.9337E+01	1.8693E+01	0.0000E+00	3.2077E+00	NaN	1.8518E+00	3.8927E+00	NaN	2.7553E+00
F3	Average	1.0641E+03	1.3194E+04	6.8340E+03	1.2464E+03	4.8506E+04	-4.5000E+02	-4.4924E+02	-4.4891E+02	-4.5000E+02	-4.4949E+02
	StandDP	4.1996E+02	3.6282E+03	2.3337E+03	1.2840E+03	1.4835E+04	0.0000E+00	1.3283E+00	8.9880E-01	0.0000E+00	8.9580E-01
	BestVal	3.0414E+02	6.7733E+03	2.5460E+03	-4.3808E+02	-4.4924E+02	-4.5000E+02	-4.5000E+02	-4.4982E+02	-4.5000E+02	-4.4999E+02
	t-Value	1.9747E+01	2.0598E+01	1.7096E+01	7.2366E+00	1.8075E+01	NaN	3.1508E+00	6.6562E+00	NaN	3.1104E+00
F7	Average	-4.4891E+02	-4.4848E+02	-4.4830E+02	-4.4998E+02	-4.4791E+02	-4.5000E+02	-4.4999E+02	-4.4997E+02	-4.4999E+02	-4.5000E+02
	StandDP	5.7380E-01	6.5360E-01	7.9260E-01	1.2000E-02	9.3556E+00	1.2000E-03	8.9000E-03	1.0600E-02	6.9000E-03	1.8000E-03
	BestVal	-4.4976E+02	-4.4941E+02	-4.4958E+02	-4.5000E+02	-4.4996E+02	-4.5000E+02	-4.5000E+02	-4.4999E+02	-4.5000E+02	-4.5000E+02
	t-Value	1.0341E+01	1.2680E+01	1.1720E+01	3.6389E+00	1.2174E+00	-5.5954E+00	1.1680E+00	8.8447E+00	NaN	-3.5610E+00
F9	Average	-4.4386E+01	-1.4460E+01	1.5202E+01	-3.0280E+01	-1.6242E+02	-1.8000E+02	-1.7996E+02	-1.2687E+02	-1.8000E+02	-1.7094E+02
	StandDP	2.4853E+01	2.7825E+01	2.1496E+01	2.3885E+01	1.5290E+01	0.0000E+00	2.1160E-01	1.7703E+01	0.0000E+00	6.3731E+00
	BestVal	-9.1755E+01	-7.5655E+01	-3.3887E+01	-8.2036E+01	-1.7970E+02	-1.8000E+02	-1.8000E+02	-1.4847E+02	-1.8000E+02	-1.8000E+02
	t-Value	2.9888E+01	3.2586E+01	4.9738E+01	3.4333E+01	6.2994E+00	NaN	1.1082E+00	1.6438E+01	NaN	7.7859E+00
F10	Average	-1.3439E+02	-1.2666E+02	-1.2984E+02	-1.4000E+02	-1.2013E+02	-1.4000E+02	-1.4000E+02	-1.4000E+02	-1.4000E+02	-1.4000E+02
	StandDP	6.4820E-01	1.1514E+00	2.0419E+00	4.4760E-10	1.6070E-01	0.0000E+00	9.4697E-04	1.7103E-06	0.0000E+00	4.7999E-07
	BestVal	-1.3547E+02	-1.2990E+02	-1.3385E+02	-1.4000E+02	-1.2042E+02	-1.4000E+02	-1.4000E+02	-1.4000E+02	-1.4000E+02	-1.4000E+02
	t-Value	4.7397E+01	6.3468E+01	2.7257E+01	1.5160E+00	6.7731E+02	NaN	3.6248E+00	7.0173E+00	NaN	1.5826E+01
F11	Average	-1.7915E+02	-1.1155E+02	-1.4227E+02	-1.7987E+02	-1.7999E+02	-1.8000E+02	-1.8000E+02	-1.8000E+02	-1.8000E+02	-1.7999E+02
	StandDP	9.3600E-02	1.8833E+01	1.0299E+01	3.0580E-01	1.4300E-02	0.0000E+00	3.2054E-04	9.6000E-03	0.0000E+00	1.1600E-02
	BestVal	-1.7942E+02	-1.3991E+02	-1.6100E+02	-1.8000E+02	-1.8000E+02	-1.8000E+02	-1.8000E+02	-1.8000E+02	-1.8000E+02	-1.8000E+02
	t-Value	4.9677E+01	1.9908E+01	2.0064E+01	2.2998E+00	3.5052E+00	NaN	1.3085E+00	2.5984E+00	NaN	2.8504E+00
Function Name	SPI	DE [48]	SCA [13]	WOA [4]	ALO [5]	SSA [6]	DA [12]	MFO [10]	BES [9]	CSA [17]	SSO [49]
F1	Average	-4.1410E+02	-5.5288E+01	-4.5000E+02	-3.4691E+02	-4.4472E+02	9.3182E+02	1.7803E+03	-4.5000E+02	-4.5000E+02	-3.7200E+02
	StandDP	7.5329E+00	4.0815E+02	3.3380E-14	1.0160E+02	5.1281E+00	1.1312E+03	3.3230E+03	0.0000E+00	0.0000E+00	3.2027E+01
	BestVal	-4.2963E+02	-4.2964E+02	-4.5000E+02	-4.4829E+02	-4.4985E+02	-2.1432E+02	-1.7808E+02	-4.5000E+02	-4.5000E+02	-4.3499E+02
	t-Value	2.6101E+01	5.2969E+00	0.0000E+00	5.5577E+00	5.6445E+00	6.6907E+00	3.6761E+00	NaN	NaN	1.3340E+01
F3	Average	4.2184E+04	1.7862E+04	5.8123E+04	6.2698E+03	2.1310E+03	1.2082E+04	2.6761E+04	-4.5000E+02	-4.5000E+02	-3.9637E+02
	StandDP	3.7443E+03	7.5044E+03	1.4733E+04	2.2170E+03	1.7357E+03	7.7223E+03	9.5474E+03	0.0000E+00	0.0000E+00	2.3535E+01
	BestVal	3.3943E+04	6.2948E+03	3.6321E+04	2.5729E+03	4.6695E+02	1.6638E+03	1.2288E+04	-4.5000E+02	-4.5000E+02	-4.3103E+02
	t-Value	6.2367E+01	1.3365E+01	2.1776E+01	1.6602E+01	8.1448E+00	8.8883E+00	1.5611E+01	NaN	NaN	1.2481E+01
F7	Average	-4.4983E+02	-4.4953E+02	-4.4999E+02	-4.4670E+02	-4.4982E+02	-4.4939E+02	-4.4683E+02	-4.5000E+02	-4.5000E+02	-3.3150E+02
	StandDP	4.0000E-02	4.5910E-01	6.8000E-03	1.7697E+00	6.7000E-02	1.1538E+00	4.7401E+00	3.9380E-05	2.1778E-04	2.0224E+01
	BestVal	-4.4989E+02	-4.4997E+02	-4.5000E+02	-4.4924E+02	-4.4993E+02	-4.4996E+02	-4.4985E+02	-4.5000E+02	-4.5000E+02	-3.7700E+02
	t-Value	2.1579E+01	5.5065E+00	-1.1720E+00	1.0186E+01	1.3730E+01	2.8606E+00	3.6493E+00	-6.6945E+00	-6.5002E+00	3.2090E+01
F9	Average	-4.6915E+01	-1.0172E+02	-1.8000E+02	-1.0479E+02	-1.3397E+02	-3.2717E+01	-1.2147E+01	-1.8000E+02	-1.8000E+02	-7.0757E+01
	StandDP	7.3232E+00	4.5966E+01	2.1111E-14	1.7933E+01	1.4411E+01	3.1461E+01	3.3111E+01	0.0000E+00	0.0000E+00	1.4810E+01
	BestVal	-6.3720E+01	-1.6706E+02	-1.8000E+02	-1.3912E+02	-1.5740E+02	-1.1929E+02	-8.9008E+01	-1.8000E+02	-1.8000E+02	-1.2118E+02
	t-Value	9.9538E+01	9.3280E+00	0.0000E+00	2.2972E+01	1.7495E+01	2.5641E+01	2.7767E+01	NaN	NaN	4.0402E+01
F10	Average	-1.3667E+02	-1.2420E+02	-1.4000E+02	-1.2435E+02	-1.3617E+02	-1.3069E+02	-1.2543E+02	-1.4000E+02	-1.4000E+02	-1.2004E+02
	StandDP	1.9890E-01	6.9692E+00	4.9510E-14	5.7768E+00	1.3026E+00	1.8920E+00	5.5561E+00	0.0000E+00	0.0000E+00	1.3000E-03
	BestVal	-1.3705E+02	-1.3688E+02	-1.4000E+02	-1.4000E+02	-1.3813E+02	-1.3451E+02	-1.3378E+02	-1.4000E+02	-1.4000E+02	-1.2004E+02
	t-Value	9.1816E+01	1.2415E+01	3.1443E+00	1.4843E+01	1.6087E+01	2.6948E+01	1.4365E+01	NaN	NaN	8.6162E+04
F11	Average	-1.7863E+02	-1.7616E+02	-1.7998E+02	-1.7824E+02	-1.7907E+02	-1.6299E+02	-1.6466E+02	-1.8000E+02	-1.8000E+02	-1.4675E+02
	StandDP	5.6200E-02	2.3475E+00	7.6100E-02	1.4168E+00	1.8500E-01	1.4040E+01	2.4040E+01	0.0000E+00	0.0000E+00	9.0212E+00
	BestVal	-1.7873E+02	-1.7886E+02	-1.8000E+02	-1.7924E+02	-1.7963E+02	-1.7612E+02	-1.7563E+02	-1.8000E+02	-1.8000E+02	-1.6454E+02
	t-Value	1.3326E+02	8.9653E+00	1.3036E+00	6.8149E+00	2.7529E+01	6.6375E+00	3.4952E+00	NaN	NaN	2.0186E+01

## Conclusion

In this paper, the first attempt to combine wavelet theory and levy flight into the PSO algorithm successfully improves the population diversity and global search for optimal solutions of PSO. By comparing the PSOLFWM with the particle swarm family algorithm and other metaheuristic algorithms proposed in recent years on a set of classical test functions, it is found that the PSOLFWM is easier to find the global optimal solution than other comparative optimization algorithms on most test functions, and has higher convergence speed. The results of statistical analysis and test further reveal that under the significance level *α* = 0.05, the PSOLFWM has significant differences with other comparison algorithms and its optimization performance is better than other comparison algorithms. Subsequently, high-dimensional function comparison test and dynamic shift comparison test were carried out. The results show that the PSOLFWM has stronger search ability than other comparison algorithms. When solving complex nonlinear dynamic problems, it can not only track the changes of the environment, but also has good stability. In the future, this method will be used to solve complex nonlinear dynamic problems, such as complex controller parameter tuning.

## Supporting information

S1 Appendix Benchmark functions. There are 21 benchmark functions in three categories are given for evaluating the proposed PSOLFWM algorithm.
(PDF)

S2 Appendix PSO family F1—F7. The numerical results of the proposed algorithm and the particle swarm family algorithms are given for the optimization of the unimodal benchmark test functions of F1-F7.
(PDF)

S3 Appendix PSO family F8—F13. The numerical results of the proposed algorithm and the eight particle swarm family algorithms are given for the optimization of the multimodal benchmark test functions of F8-F13.
(PDF)

S4 Appendix PSO family F14—F21. The numerical results of the proposed algorithm and the eight particle swarm family algorithms are given for the optimization of the others benchmark test functions of F14-F21.
(PDF)

S5 Appendix Meta-heuristic F1—F7. The numerical results of the proposed algorithm and the eight meta-heuristic algorithms are given for the optimization of the unimodal benchmark test functions of F1-F7.
(PDF)

S6 Appendix Meta-heuristic F8—F13. The numerical results of the proposed algorithm and the eight meta-heuristic algorithms are given for the optimization of the multimodal benchmark test functions of F8-F13.
(PDF)

S7 Appendix Meta-heuristic F14—F21. The numerical results of the proposed algorithm and the eight meta-heuristic algorithms are given for the optimization of the others benchmark test functions of F14-F21.
(PDF)

S8 Appendix Comparison of PSOLFWM with other algorithms using Wilcoxon’s rank sum test. Numerical results of Wilcoxon’s rank sum test for PSOLFWM and other optimization algorithms in the optimization search process for benchmark test functions F1-F21 are given.
(PDF)
