# Supplementary material for: A novel hybrid PSO based on levy flight and wavelet mutation for global optimization
Source: PLoS One. 2023 Jan 6;18(1):e0279572. doi: 10.1371/journal.pone.0279572 (PMC9821455; doi:10.1371/journal.pone.0279572)
Supplement: S1 Appendix — There are 21 benchmark functions in three categories are given for evaluating the proposed PSOLFWM algorithm. (PDF) [file pone.0279572.s001.pdf]

Table 10. Benchmark Functions

Name	Function	Dim	[Xmin,Xmax]	Fmin
Unimodal benchmark functions	$F_1(x)=\sum_{i=1}^{n} x_i^2$	30	[−100,100]	0
	$F_2(x)=\sum_{i=1}^{n}\left\lvert x_i^2\right\rvert+\prod_{i=1}^{n}\lvert x_i\rvert$	30	[−100,100]	0
	$F_3(x)=\sum_{i=1}^{n}\left(\sum_{j-1}^{i} x_j\right)^2$	30	[−100,100]	0
	$F_4(x)=\max_i\{\lvert x_i\rvert\}, 1\le i\le n$	30	[−100,100]	0
	$F_5(x)=\sum_{i}^{n-1}\left[100\left(x_{i+1}-x_i^2\right)^2+(x_i-1)^2\right]$	30	[−30,30]	0
	$F_6(x)=\sum_{i=1}^{n}\left([x_i+0.5]\right)^2$	30	[−100,100]	0
	$F_7(x)=\sum_{i=1}^{n} i x_i^4+random[0,1)$	30	[−1.28,1.28]	0
Multimodal benchmark functions	$F_8(x)=\sum_{i=1}^{n}-x_i\sin\left(\sqrt{\lvert x_i\rvert}\right)$	30	[−500,500]	−418.9829 × dim
	$F_9(x)=\sum_{i=1}^{n}\left[x_i^2-10\cos(2\pi x_i)+10\right]$	30	[−5.12,5.12]	0
	$F_{10}(x)=-20\exp\left(-0.2\sqrt{\frac{1}{n}\sum_{i=1}^{n}x_i^2}\right)-\exp\left(\frac{1}{2}\sum_{i=1}^{2}\cos(2\pi x_i)\right)+20+e$	30	[−32,32]	0
	$F_{11}(x)=\frac{1}{4000}\sum_{i=1}^{n}x_i^2-\prod_{i=1}^{n}\cos\left(\frac{x_i}{\sqrt{i}}\right)+1$	30	[−600,600]	0
	$F_{12}(x)=\frac{\pi}{n}\left\{10\sin(\pi y_1)+\sum_{i=1}^{n}(y_i-1)^2\left[1+10\sin^2(\pi y_i)\right]+(y_n-1)^2\right\}$ $y_i=1+\frac{x_i+1}{4}u(x_i,a,k,m)=\begin{cases}k(x_i-a)^m, x_i>a\\ 0,-a<x_i<a\\ k(-x_i-a)^m, x_i<-a\end{cases}$	30	[−50,50]	0
	$F_{13}=0.1\left\{\sin^2(3\pi x_1)+\sum_{i=1}^{n}(x_i-1)^2\left[1+\sin^2(3\pi x_1+1)\right]+(x_n-1)^2\left[1+\sin^2(2\pi x_n)\right]\right\}$ $+\sum_{i=1}^{n}u(x_i,5,100,4)$	30	[−50,50]	0
Fixed-Dimension multimodal benchmark functions	$F_{14}(x)=\left(\frac{1}{500}+\sum_{j=1}^{25}\frac{1}{j+\sum_{i=1}^{2}(x_i-a_{ij})^6}\right)^{-1}$	2	[−65,65]	1
	$F_{15}(x)=\sum_{i=1}^{11}\left[a_i-\frac{x_1\left(b_i^2+b_1x_2\right)}{b_1^2+b_1x_3+x_4}\right]^2$	4	[−5,5]	0.00030
	$F_{16}(x)=4x_1^2-2.1x_1^4+\frac{1}{3}x_1^6+x_1x_2-4x_2^2+4x_2^4$	2	[−5,5]	−1.0316
	$F_{17}(x)=\left(x_2-\frac{5.1}{4\pi^2}x_1^2+\frac{5}{\pi}x_1-6\right)^2+10\left(1-\frac{1}{8\pi}\right)\cos x_1+10$	2	[−5,5]	0.398
	$F_{18}(x)=\left[1+(x_1+x_2+x_3)^2\left(19-14x_1+3x_1^2-14x_2+6x_1x_2+3x_2^2\right)\right]$ $\times\left[30+(2x_1-3x_2)^2\times\left(18-32x_1+12x_2+48x_2-36x_1x_2+27x_2^2\right)\right]$	2	[−2,2]	3
	$F_{19}(x)=-\sum_{i=1}^{4}c_i\exp\left(-\sum_{j=1}^{3}a_{ij}(x_j-p_{ij})^2\right)$	3	[0,1]	−3.86
	$F_{20}(x)=-\sum_{i=1}^{4}c_i\exp\left(-\sum_{j=1}^{6}a_{ij}(x_j-p_{ij})^2\right)$	6	[0,1]	−3.32
	$F_{21}(x)=-\sum_{i=1}^{5}\left[(X-a_i)(X-a_i)^T+c_i\right]^{-1}$	4	[0,10]	−10.1532
