# Supplementary material for: A novel hybrid PSO based on levy flight and wavelet mutation for global optimization
Source: PLoS One. 2023 Jan 6;18(1):e0279572. doi: 10.1371/journal.pone.0279572 (PMC9821455; doi:10.1371/journal.pone.0279572)
Supplement: S2 Appendix — The numerical results of the proposed algorithm and the particle swarm family algorithms are given for the optimization of the unimodal benchmark test functions of F1-F7. (PDF) [file pone.0279572.s002.pdf]

# **Table 11.** PSO Family F1 - F7

| Function Name | SPI | PSO [38] | SPSO [45] | HPSOM [46] | HPSOWM [28] | BBPSO [21] | PSOLF [41] | PSOSCALF [34] | PSOGWO [47] | PSOLFWM |
|---|---|---|---|---|---|---|---|---|---|---|
| F1 | Average | 2.0117E+01 | 6.0164E+03 | 1.2194E+01 | 2.3745E-17 | 1.4794E+04 | 7.7461E-103 | 2.1848E-05 | 5.8788E-12 | **0.0000E+00** |
| | StandDP | 6.6829E+00 | 1.7808E+03 | 3.2166E+00 | 7.6727E-17 | 1.7757E+04 | 8.7618E-103 | 5.2095E-05 | 4.6208E-12 | **0.0000E+00** |
| | Med | 1.8395E+01 | 6.1560E+03 | 1.1644E+01 | 1.5256E-21 | 3.0735E+03 | 4.8836E-103 | 3.2142E-06 | 4.2409E-12 | **0.0000E+00** |
| | BestVal | 1.0305E+01 | 2.5571E+03 | 7.4449E+00 | 9.3451E-27 | 8.3120E+00 | 4.5794E-105 | 1.5523E-08 | 9.3258E-13 | **0.0000E+00** |
| | WorstVal | 3.3043E+01 | 9.2831E+03 | 1.9618E+01 | 3.3733E-16 | 4.5065E+04 | 4.2395E-102 | 2.5824E-04 | 1.9324E-11 | **0.0000E+00** |
| | Rank | 7 | 8 | 6 | 3 | 9 | 2 | 5 | 4 | **1** |
| | Average_RunTime | ***1.6500E-02*** | 2.0700E-02 | 2.1600E-02 | 2.3000E-02 | 5.3120E-01 | 1.1430E-01 | 6.0200E-02 | 7.2800E-02 | 1.1680E-01 |
| F2 | Average | 1.3940E+01 | 3.7364E+01 | 4.1961E+01 | 8.0998E-05 | 3.3440E+06 | 3.8973E-52 | 1.2000E-03 | 7.4362E-06 | **1.0963E-165** |
| | StandDP | 3.5191E+00 | 8.8996E+00 | 9.5484E+00 | 4.4235E-04 | 3.8457E+06 | 3.4645E-52 | 2.5000E-03 | 5.4334E-06 | **0.0000E+00** |
| | Med | 1.3926E+01 | 3.7017E+01 | 3.9481E+01 | 8.4145E-10 | 1.4224E+06 | 3.2250E-52 | 3.9874E-04 | 5.6638E-06 | **2.2287E-167** |
| | BestVal | 7.7979E+00 | 2.5179E+01 | 2.7001E+01 | 6.5534E-13 | 4.7938E+03 | 2.7968E-53 | 2.9825E-05 | 1.5774E-06 | **2.0244E-170** |
| | WorstVal | 2.4849E+01 | 6.2664E+01 | 6.8089E+01 | 2.4000E-03 | 1.2019E+07 | 1.5240E-51 | 1.2700E-02 | 2.4139E-05 | **2.7199E-164** |
| | Rank | 6 | 7 | 8 | 4 | 9 | 2 | 5 | 3 | **1** |
| | Average_RunTime | ***2.9800E-02*** | 3.9400E-02 | 2.9900E-02 | 4.3700E-02 | 9.7190E-01 | 1.6450E-01 | 7.6300E-02 | 1.1370E-01 | 1.7050E-01 |
| F3 | Average | 1.6409E+03 | 1.3128E+04 | 7.4302E+03 | 1.5682E+03 | 4.6804E+04 | 5.0797E-102 | 6.6900E-01 | 1.8213E+00 | **0.0000E+00** |
| | StandDP | 5.6385E+02 | 3.9519E+03 | 2.7427E+03 | 1.3910E+03 | 1.2905E+04 | 1.0776E-101 | 1.2854E+00 | 1.8465E+00 | **0.0000E+00** |
| | Med | 1.6032E+03 | 1.3667E+04 | 6.8478E+03 | 1.1457E+03 | 5.0149E+04 | 6.2119E-103 | 9.6400E-02 | 1.1423E+00 | **0.0000E+00** |
| | BestVal | 6.0710E+02 | 5.8612E+03 | 3.7744E+03 | 1.4172E+01 | 6.2498E+02 | 3.0832E-105 | 2.1000E-03 | 8.3900E-02 | **0.0000E+00** |
| | WorstVal | 2.9480E+03 | 2.0072E+04 | 1.6623E+04 | 5.4319E+03 | 6.4858E+04 | 4.7068E-101 | 4.7555E+00 | 8.5628E+00 | **0.0000E+00** |
| | Rank | 5 | 8 | 7 | 6 | 9 | 2 | 3 | 4 | **1** |
| | Average_RunTime | 1.0890E-01 | 1.6060E-01 | ***1.0780E-01*** | 1.5010E-01 | 4.6824E+00 | 2.6010E-01 | 1.6510E-01 | 2.5430E-01 | 2.7950E-01 |
| F4 | Average | 9.7577E+00 | 3.2370E+01 | 2.1585E+01 | 2.6310E-04 | 2.8512E-49 | 1.5939E-52 | 2.5000E-03 | 2.7500E-02 | **1.4348E-165** |
| | StandDP | 3.4117E+00 | 4.1808E+00 | 3.8445E+00 | 1.1000E-03 | 8.6835E-49 | 2.5161E-52 | 2.8000E-03 | 1.8300E-02 | **0.0000E+00** |
| | Med | 9.8706E+00 | 3.2688E+01 | 2.2627E+01 | 2.2758E-10 | 1.6745E-51 | 5.9335E-53 | 1.4000E-03 | 2.1600E-02 | **4.1826E-167** |
| | BestVal | 4.4525E+00 | 2.3318E+01 | 1.3355E+01 | 1.1571E-12 | 9.5096E-54 | 1.8851E-54 | 6.5384E-06 | 3.5000E-03 | **2.6727E-173** |
| | WorstVal | 1.9228E+01 | 3.8609E+01 | 2.9597E+01 | 5.8000E-03 | 3.9498E-48 | 1.0183E-51 | 1.0400E-02 | 6.9900E-02 | **3.7853E-164** |
| | Rank | 7 | 9 | 8 | 4 | 3 | 2 | 5 | 6 | **1** |
| | Average_RunTime | ***2.8600E-02*** | 3.3700E-02 | 3.7000E-02 | 3.7800E-02 | 8.4540E-01 | 1.6210E-01 | 9.6600E-02 | 1.0610E-01 | 1.6660E-01 |
| F5 | Average | 9.0051E+03 | 2.7154E+06 | 2.3243E+05 | 2.5834E+01 | 1.0433E+08 | 2.8733E+01 | 2.8612E+01 | 2.7169E+01 | **5.6530E-01** |
| | StandDP | 4.2586E+03 | 1.4513E+06 | 1.6338E+05 | 8.7585E+00 | 1.8024E+07 | 2.4120E-01 | 1.7760E-01 | 9.0960E-01 | **4.5480E-01** |
| | Med | 8.6916E+03 | 2.5408E+06 | 1.8839E+05 | 2.8704E+01 | 1.0390E+08 | 2.8855E+01 | 2.8696E+01 | 2.7022E+01 | **4.4320E-01** |
| | BestVal | 1.8992E+03 | 5.9079E+05 | 5.3465E+04 | 6.9668E-27 | 6.3623E+07 | 2.8037E+01 | 2.8015E+0 | 2.6137E+01 | **5.8900E-02** |
| | WorstVal | 2.3627E+04 | 6.6855E+06 | 7.7982E+05 | 2.8707E+01 | 1.4244E+08 | 2.8923E+01 | 2.8764E+01 | 3.0303E+01 | **1.7544E+00** |
| | Rank | 6 | 8 | 7 | 2 | 9 | 5 | 4 | 3 | **1** |
| | Average_RunTime | ***2.7300E-02*** | 3.8000E-02 | 3.0000E-02 | 3.7600E-02 | 1.0214E+00 | 1.2080E-01 | 7.1900E-02 | 9.0900E-02 | 1.7300E-01 |
| F6 | Average | 2.1747E+01 | 5.8794E+03 | 1.2733E+01 | **2.9088E-16** | 8.4539E+03 | 4.1928E+00 | 5.6340E-01 | 3.2600E-02 | 1.7000E-02 |
| | StandDP | 7.7685E+00 | 1.7693E+03 | 3.1021E+00 | **1.2784E-15** | 1.2688E+04 | 6.9020E-01 | 1.4550E-01 | 8.3900E-02 | 1.3600E-02 |
| | Med | 2.0810E+01 | 5.8153E+03 | 1.2267E+01 | **7.2619E-20** | 2.4219E+03 | 4.2774E+00 | 5.6310E-01 | 2.9881E-04 | 1.5000E-02 |
| | BestVal | 8.5402E+00 | 1.9933E+03 | 6.6421E+00 | **2.7781E-25** | 7.0813E+01 | 2.5930E+00 | 2.8200E-01 | 1.9857E-04 | 3.3000E-03 |
| | WorstVal | 4.0621E+01 | 1.0840E+04 | 1.8172E+01 | **6.8356E-15** | 4.7746E+04 | 5.8862E+00 | 8.7840E-01 | 2.6030E-01 | 6.2100E-02 |
| | Rank | 7 | 8 | 6 | **1** | 9 | 5 | 4 | 3 | 2 |
| | Average_RunTime | ***3.0900E-02*** | 3.4800E-02 | 3.6900E-02 | 4.3100E-02 | 9.1010E-01 | 1.5700E-01 | 1.0070E-01 | 1.0720E-01 | 2.2110E-01 |
| F7 | Average | 6.0089E+00 | 1.5342E+00 | 1.6348E+00 | 2.9200E-02 | 2.6970E-01 | **2.4000E-03** | 4.3000E-02 | 5.6500E-02 | **2.3000E-03** |
| | StandDP | 2.9216E+00 | 7.8100E-01 | 7.4410E-01 | 2.2500E-02 | 2.9640E-01 | **2.2000E-03** | 3.5800E-02 | 2.2500E-02 | **2.2000E-03** |
| | Med | 4.6707E+00 | 1.3941E+00 | 1.4423E+00 | 2.3200E-02 | 1.7810E-01 | **1.7000E-03** | 3.2300E-02 | 4.8100E-02 | **1.3000E-03** |
| | BestVal | 2.5181E+00 | 4.8430E-01 | 5.7810E-01 | 3.8026E-04 | 2.7100E-02 | **1.9329E-04** | 1.9000E-03 | 2.7500E-02 | **1.6291E-04** |
| | WorstVal | 1.2819E+01 | 4.0846E+00 | 3.2026E+00 | 1.0430E-01 | 1.4058E+00 | **1.0400E-02** | 1.4520E-01 | 1.1000E-01 | **9.2000E-03** |
| | Rank | 9 | 8 | 7 | 3 | 6 | **1** | 5 | 3 | **1** |
| | Average_RunTime | ***3.5800E-02*** | 3.8600E-02 | 3.6900E-02 | 3.9700E-02 | 1.4357E+00 | 8.3900E-02 | 4.8100E-02 | 8.6500E-02 | 6.8100E-02 |
