# Supplementary material for: A novel hybrid PSO based on levy flight and wavelet mutation for global optimization
Source: PLoS One. 2023 Jan 6;18(1):e0279572. doi: 10.1371/journal.pone.0279572 (PMC9821455; doi:10.1371/journal.pone.0279572)
Supplement: S3 Appendix — The numerical results of the proposed algorithm and the eight particle swarm family algorithms are given for the optimization of the multimodal benchmark test functions of F8-F13. (PDF) [file pone.0279572.s003.pdf]

Table 12. PSO Family F8 - F13

Function Name	SPI	PSO	SPSO	HPSOM	HPSOWM	BBPSO	PSOLF	PSOSCALF	PSOGWO	PSOLFWM
F8	Average	-7.1406E+03	-4.6691E+03	-6.2441E+03	-7.0356E+03	**-1.2550E+04**	-6.4706E+03	-6.3240E+03	-5.0770E+03	-1.1049E+04
	StandDP	7.3472E+02	6.1152E+02	7.4802E+02	7.5887E+02	**3.1464E+01**	5.5783E+02	1.1212E+03	1.3597E+03	1.2987E+03
	Med	-7.2163E+03	-4.6250E+03	-6.0427E+03	-7.2007E+03	**-1.2562E+04**	-6.4288E+03	-6.2452E+03	-4.7472E+03	-1.1552E+04
	BestVal	-8.6571E+03	-5.7481E+03	-7.5988E+03	-8.2503E+03	**-1.2569E+04**	-7.6728E+03	-8.8600E+03	-7.6210E+03	-1.2496E+04
	WorstVal	-5.4197E+03	-3.4973E+03	-5.0123E+03	-5.6737E+03	**-1.2410E+04**	-5.4607E+03	-4.6425E+03	-3.3900E+03	-7.9149E+03
	Rank	4	9	8	5	**1**	6	3	7	2
	Average_RunTime	***4.2600E-02***	5.7800E-02	5.1500E-02	5.6400E-02	1.5130E+00	1.5710E-01	1.3130E-01	1.2950E-01	1.9620E-01
F9	Average	1.8578E+02	1.7240E+02	1.9660E+02	1.5944E+02	9.1334E+01	**0.0000E+00**	1.0165E+01	7.7116E+01	**0.0000E+00**
	StandDP	2.2183E+01	2.6794E+01	2.0528E+01	1.7114E+01	1.0745E+02	**0.0000E+00**	1.3599E+01	2.5925E+01	**0.0000E+00**
	Med	1.8368E+02	1.7037E+02	1.9726E+02	1.5960E+02	4.9488E+01	**0.0000E+00**	4.9686E+00	7.0476E+01	**0.0000E+00**
	BestVal	1.4528E+02	1.2253E+02	1.5665E+02	1.3128E+02	2.0690E-01	**0.0000E+00**	5.8000E-03	4.7384E+01	**0.0000E+00**
	WorstVal	2.3393E+02	2.2469E+02	2.3846E+02	1.9617E+02	3.6965E+02	**0.0000E+00**	5.3628E+01	1.6248E+02	**0.0000E+00**
	Rank	6	8	5	4	9	**1**	3	7	**1**
	Average_RunTime	1.3200E-02	1.7800E-02	***1.2700E-02***	1.5700E-02	4.7040E-01	5.6200E-02	2.6300E-02	4.2200E-02	1.3860E-01
F10	Average	5.6821E+00	1.3369E+01	1.0225E+01	9.2907E-10	8.1000E-03	**8.8818E-16**	6.3091E-04	2.1759E-06	**8.8818E-16**
	StandDP	7.8660E-01	1.1081E+00	1.6642E+00	2.7248E-09	8.3000E-03	**0.0000E+00**	5.6663E-04	1.5087E-06	**0.0000E+00**
	Med	5.5725E+00	1.3214E+01	1.0138E+01	3.6828E-11	5.7000E-03	**8.8818E-16**	5.4770E-04	1.7548E-06	**8.8818E-16**
	BestVal	4.2200E+00	1.0773E+01	7.0003E+00	7.9936E-15	1.3373E-05	**8.8818E-16**	4.6889E-05	3.6562E-07	**8.8818E-16**
	WorstVal	7.6006E+00	1.5768E+01	1.4269E+01	1.3714E-08	3.5600E-02	**8.8818E-16**	2.9000E-03	6.2206E-06	**8.8818E-16**
	Rank	7	8	9	3	6	**1**	5	4	**1**
	Average_RunTime	***3.7600E-02***	4.8200E-02	3.8600E-02	4.4100E-02	1.2786E+00	1.4460E-01	8.4800E-02	1.1410E-01	3.7960E-01
F11	Average	1.1080E+00	6.9529E+01	5.8350E+01	1.0960E-01	**0.0000E+00**	**0.0000E+00**	2.8610E-01	2.6800E-02	**0.0000E+00**
	StandDP	4.0100E-02	1.6661E+01	1.6600E+01	2.8160E-01	**0.0000E+00**	**0.0000E+00**	3.2840E-01	2.3600E-02	**0.0000E+00**
	Med	1.1037E+00	6.8083E+01	5.8966E+01	1.5176E-04	**0.0000E+00**	**0.0000E+00**	1.1840E-01	3.5200E-02	**0.0000E+00**
	BestVal	1.0335E+00	3.7501E+01	2.8855E+01	6.3678E-09	**0.0000E+00**	**0.0000E+00**	3.0000E-03	4.9307E-07	**0.0000E+00**
	WorstVal	1.2205E+00	1.0367E+02	1.0085E+02	9.7340E-01	**0.0000E+00**	**0.0000E+00**	1.0045E+00	6.7500E-02	**0.0000E+00**
	Rank	5	9	8	6	**1**	**1**	7	4	**1**
	Average_RunTime	2.8500E-02	3.0300E-02	2.7400E-02	***2.7300E-02***	5.9820E-01	7.0500E-02	3.6400E-02	5.7000E-02	1.4790E-01
F12	Average	9.8979E+00	1.8522E+05	3.9658E+01	**1.5635E-10**	2.0446E+08	4.5890E-01	2.5200E-02	1.3900E-02	1.6212E-04
	StandDP	4.5897E+00	2.7347E+05	5.3277E+01	**5.3078E-10**	4.2836E+07	1.7660E-01	8.9000E-03	2.1800E-02	9.9492E-05
	Med	9.4890E+00	4.9776E+04	2.3540E+01	**2.4926E-15**	2.0597E+08	4.1490E-01	2.3700E-02	7.6000E-03	1.4466E-04
	BestVal	4.1004E+00	1.4825E+02	7.6350E+00	**4.1466E-20**	1.0927E+08	1.9810E-01	1.1500E-02	1.4120E-05	2.1542E-05
	WorstVal	1.9746E+01	1.1279E+06	2.3529E+02	**2.4067E-09**	2.7331E+08	9.6360E-01	4.9300E-02	1.2200E-01	4.1301E-04
	Rank	6	8	7	**1**	9	5	3	4	2
	Average_RunTime	***1.9680E-01***	2.9920E-01	2.0260E-01	3.1340E-01	8.8107E+00	3.2940E-01	2.5640E-01	4.4090E-01	3.2820E-01
F13	Average	2.5498E+01	2.4189E+06	5.3233E+02	**1.8808E-12**	4.4606E+08	2.7069E+00	3.6500E-01	7.1300E-02	1.1000E-03
	StandDP	1.6072E+01	2.4566E+06	6.8190E+02	**9.0733E-12**	8.4347E+07	3.0230E-01	1.2410E-01	1.0410E-01	8.1354E-04
	Med	1.8996E+01	1.6131E+06	1.9898E+02	**2.4786E-16**	4.4160E+08	2.7862E+00	3.4890E-01	6.1000E-03	9.2433E-04
	BestVal	5.2661E+00	4.3367E+04	3.5444E+01	**5.1428E-23**	2.8566E+08	1.4846E+00	1.6390E-01	2.3150E-04	1.0407E-04
	WorstVal	7.2215E+01	1.0669E+07	2.7799E+03	**4.9763E-11**	6.1872E+08	2.9891E+00	6.2690E-01	3.4180E-01	3.9000E-03
	Rank	6	8	7	**1**	9	5	4	3	2
	Average_RunTime	***8.8100E-02***	1.3780E-01	9.3300E-02	1.2830E-01	3.5286E+00	1.4730E-01	1.0120E-01	1.9000E-01	1.3370E-01
