# Supplementary material for: A novel hybrid PSO based on levy flight and wavelet mutation for global optimization
Source: PLoS One. 2023 Jan 6;18(1):e0279572. doi: 10.1371/journal.pone.0279572 (PMC9821455; doi:10.1371/journal.pone.0279572)
Supplement: S4 Appendix — The numerical results of the proposed algorithm and the eight particle swarm family algorithms are given for the optimization of the others benchmark test functions of F14-F21. (PDF) [file pone.0279572.s004.pdf]

Table 13. PSO Family F14 - F21

Function Name	SPI	PSO	SPSO	HPSOM	HPSOWM	BBPSO	PSOLF	PSOSCALF	PSOGWO	PSOLFWM
F14	Average	1.0641E+00	6.3905E+00	3.3313E+00	3.1703E+00	9.9800E-01	1.6433E+00	1.1576E+00	1.8144E+00	**9.9800E-01**
	StandDP	3.6220E-01	3.6437E+00	2.5576E+00	2.1825E+00	4.7325E-05	9.9990E-01	4.4790E-01	2.4885E+00	**1.6262E-06**
	Med	9.9800E-01	6.9033E+00	1.9920E+00	1.9920E+00	9.9800E-01	1.0090E+00	9.9800E-01	9.9800E-01	**9.9800E-01**
	BestVal	9.9800E-01	9.9800E-01	9.9800E-01	9.9800E-01	9.9800E-01	9.9800E-01	9.9800E-01	9.9800E-01	**9.9800E-01**
	WorstVal	2.9821E+00	1.3619E+01	1.0763E+01	7.8740E+00	9.9830E-01	4.8408E+00	2.9821E+00	1.0763E+01	**9.9800E-01**
	Rank	3	9	8	6	2	5	4	7	**1**
	Average_RunTime	***3.2580E-01***	3.3700E-01	3.2670E-01	3.3520E-01	1.5528E+01	3.8170E-01	3.7050E-01	6.1510E-01	3.6950E-01
F15	Average	8.7148E-04	2.3000E-03	2.5000E-03	4.2003E-04	1.6000E-03	7.1505E-04	5.5183E-04	**4.5122E-04**	4.0905E-04
	StandDP	5.3655E-04	5.1000E-03	3.3000E-03	2.4926E-04	1.1546E-04	3.7026E-04	1.4533E-04	**9.1950E-05**	9.3527E-05
	Med	7.7908E-04	8.8574E-04	1.4000E-03	3.2281E-04	1.7000E-03	5.8884E-04	5.5866E-04	**4.4090E-04**	3.6204E-04
	BestVal	4.4280E-04	3.2727E-04	5.3538E-04	3.0755E-04	1.1000E-03	3.1573E-04	3.4029E-04	**3.0985E-04**	3.1563E-04
	WorstVal	3.5000E-03	2.1700E-02	1.5200E-02	1.3000E-03	1.7000E-03	1.6000E-03	8.0180E-04	**6.4755E-04**	7.4541E-04
	Rank	7	9	8	5	3	6	4	**1**	2
	Average_RunTime	***2.6400E-02***	3.5700E-02	2.6600E-02	3.3100E-02	1.0955E+00	9.6400E-02	7.9200E-02	5.4100E-02	8.8800E-02
F16	Average	-1.0316E+00	**-1.0316E+00**	-1.0316E+00	-1.0316E+00	-1.0180E+00	-1.0316E+00	-1.0315E+00	-1.0316E+00	-1.0305E+00
	StandDP	1.1481E-06	**6.1849E-16**	9.7312E-06	5.8604E-09	1.6700E-02	7.0739E-07	1.0345E-04	3.3098E-05	9.2301E-04
	Med	-1.0316E+00	**-1.0316E+00**	-1.0316E+00	-1.0316E+00	-1.0226E+00	-1.0316E+00	-1.0316E+00	-1.0316E+00	-1.0307E+00
	BestVal	-1.0316E+00	**-1.0316E+00**	-1.0316E+00	-1.0316E+00	-1.0312E+00	-1.0316E+00	-1.0316E+00	-1.0316E+00	-1.0316E+00
	WorstVal	-1.0316E+00	**-1.0316E+00**	-1.0316E+00	-1.0316E+00	-9.5720E-01	-1.0316E+00	-1.0313E+00	-1.0315E+00	-1.0283E+00
	Rank	4	**1**	5	2	9	3	7	6	8
	Average_RunTime	***2.0500E-02***	2.0600E-02	2.1600E-02	2.5200E-02	8.7860E-01	7.0100E-02	6.2300E-02	4.1400E-02	6.6000E-02
F17	Average	3.9790E-01	**3.9790E-01**	3.9790E-01	3.9790E-01	4.0810E-01	3.9790E-01	3.9820E-01	3.9790E-01	3.9880E-01
	StandDP	2.5596E-07	**0.0000E+00**	1.8446E-06	1.3850E-09	8.0000E-03	1.0388E-05	4.0676E-04	4.6659E-06	1.0000E-03
	Med	3.9790E-01	**3.9790E-01**	3.9790E-01	3.9790E-01	4.0410E-01	3.9790E-01	3.9810E-01	3.9790E-01	3.9830E-01
	BestVal	3.9790E-01	**3.9790E-01**	3.9790E-01	3.9790E-01	3.9930E-01	3.9790E-01	3.9790E-01	3.9790E-01	3.9790E-01
	WorstVal	3.9790E-01	**3.9790E-01**	3.9790E-01	3.9790E-01	4.2510E-01	3.9790E-01	3.9950E-01	3.9790E-01	4.0160E-01
	Rank	3	**1**	4	2	9	6	7	5	8
	Average_RunTime	***6.3000E-03***	6.5000E-03	7.1000E-03	8.2000E-03	2.3590E-01	2.9800E-02	2.3400E-02	1.1600E-02	2.1400E-02
F18	Average	3.0000E+00	**3.0000E+00**	3.0000E+00	3.0000E+00	3.1580E+00	3.0000E+00	3.0011E+00	3.0000E+00	3.0444E+00
	StandDP	5.5762E-05	**1.9654E-15**	1.8618E-05	1.9883E-07	1.4470E-01	2.2719E-05	1.6000E-03	2.2913E-06	3.7800E-02
	Med	3.0000E+00	**3.0000E+00**	3.0000E+00	3.0000E+00	3.1062E+00	3.0000E+00	3.0003E+00	3.0000E+00	3.0363E+00
	BestVal	3.0000E+00	**3.0000E+00**	3.0000E+00	3.0000E+00	3.0150E+00	3.0000E+00	3.0000E+00	3.0000E+00	3.0000E+00
	WorstVal	3.0002E+00	**3.0000E+00**	3.0001E+00	3.0000E+00	3.6451E+00	3.0001E+00	3.0059E+00	3.0000E+00	3.1299E+00
	Rank	6	**1**	4	2	9	5	7	3	8
	Average_RunTime	***1.6000E-02***	1.6000E-02	1.7500E-02	1.9700E-02	6.0530E-01	6.5200E-02	5.5400E-02	2.7900E-02	5.4700E-02
F19	Average	-3.8603E+00	-3.8628E+00	-3.8625E+00	**-3.8628E+00**	-3.8530E+00	-3.8620E+00	-3.8593E+00	-3.8628E+00	-3.8566E+00
	StandDP	2.3000E-03	2.4186E-06	1.2000E-03	**2.2516E-07**	7.3000E-03	1.1000E-03	3.9000E-03	1.7257E-05	5.5000E-03
	Med	-3.8611E+00	-3.8628E+00	-3.8628E+00	**-3.8628E+00**	-3.8554E+00	-3.8624E+00	-3.8609E+00	-3.8628E+00	-3.8579E+00
	BestVal	-3.8628E+00	-3.8628E+00	-3.8628E+00	**-3.8628E+00**	-3.8616E+00	-3.8628E+00	-3.8627E+00	-3.8628E+00	-3.8628E+00
	WorstVal	-3.8532E+00	-3.8628E+00	-3.8561E+00	**-3.8628E+00**	-3.8338E+00	-3.8581E+00	-3.8462E+00	-3.8627E+00	-3.8386E+00
	Rank	6	2	5	**1**	9	4	7	3	8
	Average_RunTime	***2.4500E-02***	2.5500E-02	2.5300E-02	2.8400E-02	1.0379E+00	7.8600E-02	7.2000E-02	4.6600E-02	7.4700E-02
F20	Average	-3.1315E+00	-3.2424E+00	-3.2192E+00	-3.2242E+00	-3.0022E+00	**-3.2876E+00**	-3.2039E+00	-3.2462E+00	-3.1934E+00
	StandDP	5.8500E-02	7.9200E-02	9.5100E-02	7.8400E-02	9.5300E-02	**5.7000E-02**	7.2800E-02	5.8600E-02	9.0800E-02
	Med	-3.1254E+00	-3.2015E+00	-3.1965E+00	-3.1935E+00	-2.9931E+00	**-3.3213E+00**	-3.1708E+00	-3.2029E+00	-3.2147E+00
	BestVal	-3.2787E+00	-3.3220E+00	-3.3213E+00	-3.3220E+00	-3.2749E+00	**-3.3218E+00**	-3.3106E+00	-3.3220E+00	-3.2984E+00
	WorstVal	-3.0326E+00	-3.0867E+00	-3.0688E+00	-3.1035E+00	-2.8555E+00	**-3.1723E+00**	-3.1113E+00	-3.2009E+00	-2.9344E+00
	Rank	8	3	5	4	9	**1**	6	2	7
	Average_RunTime	2.7000E-02	3.6300E-02	***2.6400E-02***	2.9100E-02	1.0611E+00	8.5400E-02	7.3700E-02	5.5800E-02	8.8800E-02
F21	Average	-8.3932E+00	-5.3102E+00	-7.4872E+00	-7.1384E+00	**-1.0151E+01**	-8.6967E+00	-8.6662E+00	-9.6248E+00	-9.7976E+00
	StandDP	3.0190E+00	3.1407E+00	3.2851E+00	3.1837E+00	**4.2000E-03**	2.6252E+00	1.6419E+00	1.5389E+00	2.8350E-01
	Med	-1.0150E+01	-5.0918E+00	-9.9720E+00	-7.6270E+00	**-1.0153E+01**	-9.9622E+00	-9.2487E+00	-1.0147E+01	-9.8942E+00
	BestVal	-1.0153E+01	-1.0153E+01	-1.0153E+01	-1.0153E+01	**-1.0153E+01**	-1.0135E+01	-1.0077E+01	-1.0152E+01	-1.0128E+01
	WorstVal	-2.6295E+00	-2.6305E+00	-2.6173E+00	-2.6299E+00	**-1.0133E+01**	-2.6302E+00	-4.9404E+00	-5.0933E+00	-8.8159E+00
	Rank	6	7	9	8	**1**	5	4	3	2
	Average_RunTime	***2.3400E-02***	2.6500E-02	2.4700E-02	2.9300E-02	1.0891E+00	7.2700E-02	6.2300E-02	4.7800E-02	7.2400E-02
