# Supplementary material for: A novel hybrid PSO based on levy flight and wavelet mutation for global optimization
Source: PLoS One. 2023 Jan 6;18(1):e0279572. doi: 10.1371/journal.pone.0279572 (PMC9821455; doi:10.1371/journal.pone.0279572)
Supplement: S5 Appendix — The numerical results of the proposed algorithm and the eight meta-heuristic algorithms are given for the optimization of the unimodal benchmark test functions of F1-F7. (PDF) [file pone.0279572.s005.pdf]

Table 14. Meta-heuristic F1 - F7

Function Name	SPI	PSOLFWM	GWO [3]	DE [48]	SCA [13]	WOA [4]	ALO [5]	SS [6]	DA [12]	MFO [10]	BES [9]	CSA [17]	SSO [49]
F1	Average	**0.0000E+00**	3.2155E-11	3.8012E+01	4.3457E+02	1.0318E-32	1.3166E+02	3.7687E+00	1.6447E+03	2.0067E+03	**0.0000E+00**	1.5903E-126	1.9223E-33
	StandDP	**0.0000E+00**	2.4335E-11	8.5119E+00	4.5477E+02	2.4659E-32	1.7663E+02	2.3732E+00	1.4871E+03	2.9829E+03	**0.0000E+00**	6.1155E-126	8.8546E-33
	Med	**0.0000E+00**	2.2263E-11	3.5512E+01	1.9816E+02	3.7729E-34	3.7701E+01	3.2410E+00	1.3666E+03	1.1135E+03	**0.0000E+00**	1.5501E-127	2.4472E-38
	BestVal	**0.0000E+00**	3.4658E-12	2.4542E+01	2.2800E+01	4.2044E-38	4.6086E+00	3.6910E-01	9.3949E+01	2.6288E+02	**0.0000E+00**	2.7780E-129	5.0070E-46
	WorstVal	**0.0000E+00**	1.1085E-10	6.1978E+01	1.5152E+03	1.0569E-31	7.7857E+02	7.7924E+00	7.9024E+03	1.0936E+04	**1.0936E+04**	1.0936E+04	4.8251E-32
	Rank	**1**	6	10	9	5	8	7	11	12	**1**	3	4
	Average_RunTime	1.5130E-01	7.5800E-02	2.2490E-01	6.1700E-02	***3.6200E-02***	3.2119E+00	7.2100E-02	1.3604E+01	1.3666E+01	4.9070E-01	3.2920E-01	6.0360E-01
F2	Average	**1.1579E-166**	2.9942E-07	1.7482E+00	9.9510E-01	3.5419E-21	6.2470E+01	5.0047E+00	1.3898E+01	3.6998E+01	**0.0000E+00**	2.6395E-66	5.9203E-22
	StandDP	**0.0000E+00**	1.5537E-07	2.0020E-01	6.4170E-01	1.1310E-20	4.6931E+01	2.0930E+00	5.3096E+00	1.6924E+01	**0.0000E+00**	2.5094E-66	2.6135E-21
	Med	**1.9697E-168**	2.6282E-07	1.7144E+00	7.8330E-01	1.8438E-22	5.1436E+01	5.0336E+00	1.3317E+01	3.5095E+01	**0.0000E+00**	1.7560E-66	3.1485E-24
	BestVal	**3.8199E-170**	6.6536E-08	1.4629E+00	2.1470E-01	1.7873E-24	9.6051E+00	1.1885E+00	6.2094E+00	1.1502E+01	**0.0000E+00**	2.7153E-67	8.2093E-29
	WorstVal	**2.6374E-165**	8.2169E-07	2.3361E+00	2.7353E+00	5.2735E-20	1.9388E+02	1.1430E+01	2.5736E+01	7.2544E+01	**7.2544E+01**	7.2544E+01	1.4295E-20
	Rank	**1**	6	7	8	5	12	9	10	11	**1**	3	4
	Average_RunTime	1.1590E-01	6.4900E-02	2.0660E-01	5.0600E-02	***2.7400E-02***	3.1803E+00	6.3000E-02	9.1059E+00	9.1572E+00	4.5390E-01	2.9550E-01	5.6320E-01
F3	Average	**0.0000E+00**	2.7820E-01	4.1143E+04	1.5313E+04	6.4232E+04	6.0483E+03	1.8574E+03	1.0131E+04	2.5310E+04	**0.0000E+00**	5.6760E-103	1.6937E-09
	StandDP	**0.0000E+00**	3.2720E-01	4.7868E+03	8.4759E+03	1.6138E+04	3.0130E+03	7.7920E+02	6.3089E+03	7.8274E+03	**0.0000E+00**	2.1308E-102	7.6923E-09
	Med	**0.0000E+00**	1.6720E-01	4.1029E+04	1.4448E+04	6.2049E+04	4.8437E+03	1.7335E+03	9.7358E+03	2.4318E+04	**0.0000E+00**	3.8710E-105	6.1123E-14
	BestVal	**0.0000E+00**	1.0600E-02	3.2297E+04	5.3286E+03	4.0512E+04	2.6816E+03	6.8483E+02	9.8885E+02	1.0974E+04	**0.0000E+00**	1.6835E-108	3.7074E-22
	WorstVal	**0.0000E+00**	1.2966E+00	5.0775E+04	4.8608E+04	1.0735E+05	1.4776E+04	3.4278E+03	2.3302E+04	3.9964E+04	**3.9964E+04**	3.9964E+04	4.1586E-08
	Rank	**1**	5	11	9	12	8	6	7	10	**1**	3	4
	Average_RunTime	2.7380E-01	1.3980E-01	3.5870E-01	1.3060E-01	***1.0540E-01***	3.3510E+00	1.4220E-01	1.0558E+01	1.0688E+01	7.1600E-01	5.3700E-01	6.4290E-01
F4	Average	**2.5085E-166**	7.4000E-03	3.9281E+01	5.1384E+01	3.8022E+01	1.8915E+01	1.1336E+01	2.7862E+01	6.0629E+01	**0.0000E+00**	2.3263E-58	3.4643E-11
	StandDP	**0.0000E+00**	3.7000E-03	3.8088E+00	9.1041E+00	2.8701E+01	3.6068E+00	3.8410E+00	7.3605E+00	9.2694E+00	**0.0000E+00**	3.4586E-58	1.7266E-10
	Med	**1.7765E-167**	6.9000E-03	3.8875E+01	5.3528E+01	2.8828E+01	1.8001E+01	1.0976E+01	2.8212E+01	6.2983E+01	**0.0000E+00**	6.6854E-59	6.6183E-14
	BestVal	**6.1702E-172**	1.6000E-03	3.2248E+01	2.5480E+01	5.8340E-01	1.3669E+01	5.3270E+00	1.2036E+01	3.8759E+01	**0.0000E+00**	5.7073E-60	2.1093E-23
	WorstVal	**1.4165E-165**	1.5300E-02	4.9168E+01	6.9455E+01	8.0956E+01	2.6242E+01	2.5793E+01	4.3920E+01	7.2870E+01	**7.2870E+01**	7.2870E+01	9.4751E-10
	Rank	**1**	5	7	10	12	6	8	9	11	**1**	3	4
	Average_RunTime	1.1250E-01	6.2300E-02	1.9360E-01	4.9800E-02	***2.5300E-02***	3.1754E+00	6.1200E-02	8.5236E+00	8.5734E+00	4.4680E-01	2.9280E-01	5.5540E-01
F5	Average	**4.0040E-01**	2.7345E+01	5.8588E+03	5.0874E+05	2.8366E+01	4.4621E+03	9.2426E+02	1.4942E+05	5.0685E+05	2.0643E+01	2.6184E+01	2.8099E+01
	StandDP	**2.7990E-01**	7.3750E-01	1.9803E+03	6.8036E+05	3.5860E-01	5.6738E+03	1.0345E+03	1.5260E+05	4.0563E+05	7.1180E-01	1.2140E-01	4.1070E-01
	Med	**3.6290E-01**	2.7138E+01	5.3496E+03	3.3199E+05	2.8444E+01	2.3450E+03	4.9934E+02	8.9648E+04	4.3375E+05	2.0483E+01	2.6220E+01	2.8084E+01
	BestVal	**7.4000E-03**	2.5909E+01	2.6961E+03	3.6137E+03	2.7731E+01	3.3632E+02	7.0005E+01	3.0612E+03	4.6070E+04	1.9380E+01	2.5944E+01	2.7283E+01
	WorstVal	**1.1394E+00**	2.8774E+01	1.1731E+04	3.6177E+06	2.8792E+01	2.4873E+04	4.5453E+03	6.0831E+05	1.4992E+06	1.4992E+06	1.4992E+06	2.8915E+01
	Rank	**1**	3	9	11	6	8	7	10	12	2	4	5
	Average_RunTime	1.7640E-01	7.1900E-02	2.2620E-01	6.0100E-02	***3.5700E-02***	3.2060E+00	7.1200E-02	8.8072E+00	8.8674E+00	4.9850E-01	3.2320E-01	5.9430E-01
F6	Average	1.0800E-02	7.7330E-01	4.1591E+01	3.8268E+02	5.9670E-01	8.6318E+01	4.4608E+00	1.7617E+03	1.9445E+03	**3.1437E-12**	2.6400E-07	5.0694E+00
	StandDP	8.4000E-03	4.2240E-01	1.0462E+01	2.9199E+02	2.4240E-01	1.0903E+02	3.1164E+00	1.1336E+03	2.9639E+03	**7.3773E-12**	2.0569E-07	2.3680E-01
	Med	7.9000E-03	7.5220E-01	4.1730E+01	2.4546E+02	6.1420E-01	3.9328E+01	3.9211E+00	1.6068E+03	9.5185E+02	**6.3732E-13**	2.1663E-07	5.0565E+00
	BestVal	1.6000E-03	2.4130E-01	2.1816E+01	3.7787E+01	1.3480E-01	3.1792E+00	6.2230E-01	2.2005E+02	5.1793E+02	**1.5252E-14**	1.6448E-08	4.5805E+00
	WorstVal	3.1800E-02	1.7821E+00	6.9721E+01	9.9284E+02	1.2450E+00	4.6139E+02	1.1403E+01	5.3105E+03	1.0838E+04	**1.0838E+04**	1.0838E+04	5.6120E+00
	Rank	3	6	8	10	5	9	7	11	12	**1**	2	4
	Average_RunTime	1.4300E-01	6.2400E-02	2.0640E-01	5.0700E-02	***2.5900E-02***	3.2454E+00	6.1400E-02	8.7838E+00	8.8347E+00	4.7360E-01	2.9330E-01	5.6140E-01
F7	Average	1.5000E-03	4.5000E-03	1.8660E-01	6.5690E-01	6.7000E-03	4.3250E-01	2.0690E-01	5.9030E-01	1.4144E+00	**6.6003E-05**	1.7290E-04	3.1852E-04
	StandDP	1.4000E-03	1.9000E-03	4.4900E-02	6.8230E-01	6.3000E-03	1.7250E-01	7.4200E-02	3.8410E-01	1.4980E+00	**4.0190E-05**	1.6211E-04	5.2286E-04
	Med	8.2987E-04	4.4000E-03	1.7600E-01	3.4600E-01	5.1000E-03	4.2820E-01	2.0590E-01	4.7700E-01	8.1800E-01	**6.4362E-05**	1.1979E-04	1.5487E-04
	BestVal	1.6184E-05	5.3364E-04	8.5200E-02	8.4700E-02	2.2214E-04	1.8990E-01	7.6900E-02	1.3180E-01	2.0260E-01	**3.9231E-06**	1.1132E-05	2.1871E-05
	WorstVal	4.4000E-03	9.2000E-03	2.7810E-01	2.4246E+00	2.6000E-02	8.8390E-01	3.7250E-01	1.3521E+00	5.8108E+00	**5.8108E+00**	5.8108E+00	2.9000E-03
	Rank	3	6	9	8	5	11	7	10	12	**1**	2	4
	Average_RunTime	1.2060E-01	1.0440E-01	2.8710E-01	9.1800E-02	***6.8200E-02***	3.2654E+00	1.0450E-01	8.6969E+00	8.7907E+00	5.9420E-01	4.2880E-01	6.1550E-01
