# Supplementary material for: A novel hybrid PSO based on levy flight and wavelet mutation for global optimization
Source: PLoS One. 2023 Jan 6;18(1):e0279572. doi: 10.1371/journal.pone.0279572 (PMC9821455; doi:10.1371/journal.pone.0279572)
Supplement: S6 Appendix — The numerical results of the proposed algorithm and the eight meta-heuristic algorithms are given for the optimization of the multimodal benchmark test functions of F8-F13. (PDF) [file pone.0279572.s006.pdf]

Table 15. Meta-heuristic F8 - F13

Function Name	SPI	PSOLFWM	GWO	DE	SCA	WOA	ALO	SSA	DA	MFO	BES	CSA	SSO
F8	Average	-1.0801E+04	-5.8348E+03	-7.4775E+03	-3.7342E+03	**-1.0308E+04**	-5.7732E+03	-7.5638E+03	-5.9591E+03	-8.4232E+03	-5.6928E+03	-8.4723E+03	-5.7406E+03
	StandDP	1.3843E+03	9.6332E+02	3.2602E+02	3.4369E+02	**1.7744E+03**	1.0689E+03	8.3749E+02	7.1822E+02	8.2296E+02	1.1459E+03	2.3268E+03	9.6209E+01
	Med	-1.0931E+04	-6.0917E+03	-7.4283E+03	-3.6383E+03	**-9.7759E+03**	-5.4177E+03	-7.6688E+03	-6.0414E+03	-8.4605E+03	-5.3919E+03	-9.4116E+03	-5.7132E+03
	BestVal	-1.2500E+04	-7.4159E+03	-8.2916E+03	-4.4330E+03	**-1.2568E+04**	-1.0288E+04	-9.4599E+03	-7.1684E+03	-9.8182E+03	-9.1879E+03	-1.0571E+04	-5.9542E+03
	WorstVal	-5.7292E+03	-3.1895E+03	-7.0281E+03	-3.2455E+03	**-7.1266E+03**	-5.4177E+03	-5.6207E+03	-4.1910E+03	-6.6720E+03	-4.2830E+03	-3.1477E+03	-5.6503E+03
	Rank	2	9	8	12	**1**	4	6	10	5	7	3	11
	Average_RunTime	4.6390E-01	1.8660E-01	5.5250E-01	1.5120E-01	***8.8700E-02***	1.2872E+01	1.7890E-01	3.4069E+01	3.4217E+01	1.2076E+00	7.9720E-01	1.4563E+00
F9	Average	**0.0000E+00**	1.3678E+01	1.2893E+02	8.9751E+01	5.6843E-15	7.6246E+01	4.0133E+01	1.5273E+02	1.6382E+02	**0.0000E+00**	**0.0000E+00**	**0.0000E+00**
	StandDP	**0.0000E+00**	1.4531E+01	1.1086E+01	4.5060E+01	2.2884E-14	2.8479E+01	1.3293E+01	3.5435E+01	3.4677E+01	**0.0000E+00**	**0.0000E+00**	**0.0000E+00**
	Med	**0.0000E+00**	1.0705E+01	1.3198E+02	8.8043E+01	0.0000E+00	7.1106E+01	3.7454E+01	1.4806E+02	1.6745E+02	**0.0000E+00**	**0.0000E+00**	**0.0000E+00**
	BestVal	**0.0000E+00**	2.1869E-07	1.0809E+02	9.3694E+00	0.0000E+00	3.6096E+01	1.7533E+01	9.6795E+01	1.0569E+02	**0.0000E+00**	**0.0000E+00**	**0.0000E+00**
	WorstVal	**0.0000E+00**	8.6835E+01	1.4629E+02	2.1330E+02	1.1369E-13	1.7926E+02	6.6956E+01	2.4624E+02	2.3868E+02	**0.0000E+00**	**0.0000E+00**	**0.0000E+00**
	Rank	**1**	8	6	12	5	9	7	11	10	**1**	**1**	**1**
	Average_RunTime	2.8990E-01	6.9000E-02	2.1360E-01	5.7200E-02	***2.8800E-02***	3.2209E+00	6.8600E-02	8.9626E+00	9.0209E+00	4.4970E-01	3.0160E-01	5.5890E-01
F10	Average	**8.8818E-16**	1.2695E-06	3.4064E+00	1.5016E+01	9.5331E-15	9.4275E+00	3.3861E+00	1.0371E+01	1.4149E+01	**8.8818E-16**	**8.8818E-16**	2.0724E-15
	StandDP	**0.0000E+00**	6.9075E-07	2.5090E-01	7.5358E+00	5.3338E-15	2.3429E+00	8.1140E-01	2.7931E+00	5.1852E+00	**0.0000E+00**	**0.0000E+00**	1.7034E-15
	Med	**8.8818E-16**	1.0937E-06	3.3995E+00	2.0238E+01	7.9936E-15	9.7465E+00	3.5608E+00	1.0085E+01	1.5504E+01	**8.8818E-16**	**8.8818E-16**	8.8818E-16
	BestVal	**8.8818E-16**	3.9089E-07	3.0193E+00	1.7370E+00	4.4409E-15	4.2807E+00	1.8798E+00	4.2410E+00	6.6323E+00	**8.8818E-16**	**8.8818E-16**	8.8818E-16
	WorstVal	**8.8818E-16**	3.4447E-06	4.0272E+00	2.0376E+01	2.2204E-14	1.2872E+01	5.0867E+00	1.9523E+01	1.9947E+01	**8.8818E-16**	**8.8818E-16**	4.4409E-15
	Rank	**1**	6	7	12	5	9	8	10	11	**1**	**1**	4
	Average_RunTime	4.2680E-01	9.0700E-02	3.0970E-01	8.3300E-02	***4.3800E-02***	4.3540E+00	9.7600E-02	1.2878E+01	1.2961E+01	6.3990E-01	4.2420E-01	7.3590E-01
F11	Average	**0.0000E+00**	6.6000E-03	1.3567E+00	5.6368E+00	1.3400E-02	1.6971E+00	9.4150E-01	1.4102E+01	2.3836E+01	**0.0000E+00**	**0.0000E+00**	**0.0000E+00**
	StandDP	**0.0000E+00**	1.1400E-02	6.5100E-02	5.6820E+00	7.3400E-02	1.0924E+00	1.9460E-01	9.3696E+00	3.3143E+01	**0.0000E+00**	**0.0000E+00**	**0.0000E+00**
	Med	**0.0000E+00**	7.8012E-11	1.3540E+00	4.0509E+00	0.0000E+00	1.2998E+00	1.0260E+00	1.3036E+01	9.3609E+00	**0.0000E+00**	**0.0000E+00**	**0.0000E+00**
	BestVal	**0.0000E+00**	1.2626E-11	1.2276E+00	9.7000E-01	0.0000E+00	9.0200E-01	2.8090E-01	2.1503E+00	5.0018E+00	**0.0000E+00**	**0.0000E+00**	**0.0000E+00**
	WorstVal	**0.0000E+00**	4.2600E-02	1.5169E+00	3.2254E+01	4.0180E-01	5.1689E+00	1.2209E+00	3.7484E+01	9.8302E+01	**0.0000E+00**	**0.0000E+00**	**0.0000E+00**
	Rank	**1**	5	6	10	7	9	8	11	12	**1**	**1**	**1**
	Average_RunTime	3.1510E-01	7.8700E-02	2.4860E-01	6.9900E-02	***3.9300E-02***	3.2697E+00	8.0500E-02	8.7367E+00	8.8071E+00	5.0160E-01	3.3520E-01	5.9370E-01
F12	Average	1.5876E-04	4.0600E-02	4.2452E+00	1.0520E+06	3.6100E-02	1.9038E+01	6.4460E+00	2.3617E+04	2.2975E+05	**9.9647E-14**	7.2310E-09	5.9100E-01
	StandDP	1.1693E-04	2.3400E-02	7.6890E-01	2.5081E+06	1.9200E-02	7.8633E+00	2.2478E+00	1.1721E+05	7.1276E+05	**3.5696E-13**	7.3503E-09	5.9900E-02
	Med	1.5645E-04	3.4200E-02	4.2950E+00	2.3823E+04	3.3300E-02	1.9411E+01	6.8252E+00	2.4828E+01	7.5860E+03	**1.1044E-14**	3.9886E-09	5.9490E-01
	BestVal	1.2464E-05	1.2600E-02	2.4789E+00	5.3500E+00	1.1600E-02	5.9696E+00	2.3912E+00	3.6719E+00	2.1356E+01	**2.7684E-16**	1.0334E-09	4.6090E-01
	WorstVal	4.3587E-04	1.1660E-01	5.5952E+00	1.1005E+07	1.1250E-01	3.9440E+01	1.2126E+01	6.4101E+05	3.5406E+06	**1.9420E-12**	3.3088E-08	6.7590E-01
	Rank	3	5	7	12	4	9	8	10	11	**1**	2	6
	Average_RunTime	3.3530E-01	2.3530E-01	6.0070E-01	2.2390E-01	***1.9140E-01***	4.3132E+00	2.4900E-01	1.2692E+01	1.2922E+01	1.1239E+00	8.4470E-01	8.5090E-01
F13	Average	**1.4000E-03**	6.6430E-01	1.4191E+01	4.6021E+06	5.4360E-01	1.3067E+02	2.8255E+01	2.0171E+05	1.2801E+06	2.8353E+00	3.4200E-02	2.5686E+00
	StandDP	**1.4000E-03**	2.5090E-01	3.7578E+00	1.0078E+07	2.6400E-01	3.6889E+02	1.3058E+01	4.3062E+05	1.4849E+06	4.6690E-01	5.0300E-02	7.5800E-02
	Med	**1.1000E-03**	6.5710E-01	1.2939E+01	9.7549E+05	5.2030E-01	6.0922E+01	2.9126E+01	4.3885E+03	6.0623E+05	2.9661E+00	1.1000E-02	2.5658E+00
	BestVal	**1.5391E-04**	1.6120E-01	9.4318E+00	4.7447E+02	1.6520E-01	3.5416E+01	5.3692E+00	3.6474E+01	1.9416E+04	7.2120E-01	1.3912E-07	2.3655E+00
	WorstVal	**6.8000E-03**	1.2875E+00	2.1591E+01	5.0414E+07	1.3432E+00	2.0823E+03	4.9779E+01	1.5920E+06	5.7514E+06	2.9661E+00	1.9630E-01	2.7031E+00
	Rank	**1**	4	7	12	5	9	8	10	11	6	2	3
	Average_RunTime	2.7380E-01	1.9310E-01	4.8470E-01	1.7870E-01	***1.5260E-01***	3.4096E+00	1.9330E-01	8.9475E+00	9.1304E+00	8.6850E-01	6.7750E-01	6.9690E-01
