# Supplementary material for: A novel hybrid PSO based on levy flight and wavelet mutation for global optimization
Source: PLoS One. 2023 Jan 6;18(1):e0279572. doi: 10.1371/journal.pone.0279572 (PMC9821455; doi:10.1371/journal.pone.0279572)
Supplement: S7 Appendix — The numerical results of the proposed algorithm and the eight meta-heuristic algorithms are given for the optimization of the others benchmark test functions of F14-F21. (PDF) [file pone.0279572.s007.pdf]

Table 16. Meta-heuristic F14 - F21

Function Name	SPI	PSOLFWM	GWO	DE	SCA	WOA	ALO	SSA	DA	MFO	BES	CSA	SSO
F14	Average	9.9800E-01	3.2227E+00	**9.9800E-01**	1.6105E+00	3.0588E+00	2.5419E+00	1.4610E+00	2.1544E+00	1.4281E+00	3.2767E+00	3.1294E+00	2.7172E+00
	StandDP	3.0783E-06	3.3967E+00	**0.0000E+00**	9.1410E-01	3.3492E+00	2.4276E+00	8.5310E-01	1.2221E+00	7.6730E-01	4.1862E+00	2.9633E+00	2.3716E+00
	Med	9.9800E-01	1.9920E+00	**9.9800E-01**	1.0105E+00	9.9800E-01	1.9920E+00	9.9800E-01	1.9920E+00	9.9800E-01	9.9800E-01	2.9821E+00	2.9821E+00
	BestVal	9.9800E-01	9.9800E-01	**9.9800E-01**	9.9810E-01	9.9800E-01	9.9800E-01	9.9800E-01	9.9800E-01	9.9800E-01	9.9800E-01	9.9800E-01	9.9800E-01
	WorstVal	9.9800E-01	1.2671E+01	**9.9800E-01**	2.9821E+00	1.0763E+01	1.2671E+01	3.9683E+00	3.9683E+00	3.9683E+00	1.2671E+01	1.2671E+01	1.0763E+01
	Rank	2	11	**1**	5	10	8	4	6	3	12	9	7
	Average_RunTime	3.8070E-01	3.2220E-01	8.0750E-01	3.2000E-01	***3.1480E-01***	6.9490E-01	3.4860E-01	3.4982E+00	3.8197E+00	1.4306E+00	9.5930E-01	3.8900E-01
F15	Average	**4.2078E-04**	3.2000E-03	8.1595E-04	1.1000E-03	7.7995E-04	5.0000E-03	2.3000E-03	2.3000E-03	9.2327E-04	3.7888E-04	6.5828E-04	5.0690E-04
	StandDP	**9.8647E-05**	6.9000E-03	1.1678E-04	3.5450E-04	4.2263E-04	1.3100E-02	5.0000E-03	3.5000E-03	3.5734E-04	2.3638E-04	1.1831E-04	2.4361E-04
	Med	**3.9415E-04**	5.4444E-04	8.0204E-04	8.9621E-04	6.9572E-04	1.3000E-03	8.1833E-04	1.7000E-03	7.8642E-04	3.0749E-04	7.1064E-04	4.6943E-04
	BestVal	**3.0844E-04**	3.0992E-04	6.2314E-04	6.1088E-04	3.1086E-04	4.2886E-04	3.2141E-04	7.6572E-04	3.0874E-04	3.0749E-04	3.3591E-04	3.3143E-04
	WorstVal	**6.8577E-04**	2.0400E-02	1.2000E-03	1.7000E-03	2.0000E-03	6.9100E-02	2.0600E-02	2.0400E-02	2.3000E-03	1.2000E-03	7.5237E-04	1.7000E-03
	Rank	**1**	11	2	6	8	12	10	9	7	4	3	5
	Average_RunTime	5.9000E-02	2.8300E-02	1.6130E-01	2.5300E-02	***2.4200E-02***	5.4600E-01	4.3300E-02	7.5336E+00	7.5613E+00	4.4030E-01	8.9600E-02	1.1010E-01
F16	Average	-1.0303E+00	-1.0316E+00	**-1.0316E+00**	-1.0315E+00	-1.0316E+00	-1.0316E+00	-1.0316E+00	-1.0316E+00	-1.0316E+00	-1.0316E+00	-1.0316E+00	-1.0313E+00
	StandDP	1.3000E-03	7.5016E-08	**6.4539E-16**	7.5775E-05	2.3248E-09	1.4845E-13	5.4862E-14	1.9046E-09	6.5843E-16	3.0271E-08	4.6677E-06	3.5960E-04
	Med	-1.0308E+00	-1.0316E+00	**-1.0316E+00**	-1.0316E+00	-1.0316E+00	-1.0316E+00	-1.0316E+00	-1.0316E+00	-1.0316E+00	-1.0316E+00	-1.0316E+00	-1.0314E+00
	BestVal	-1.0315E+00	-1.0316E+00	**-1.0316E+00**	-1.0316E+00	-1.0316E+00	-1.0316E+00	-1.0316E+00	-1.0316E+00	-1.0316E+00	-1.0316E+00	-1.0316E+00	-1.0316E+00
	WorstVal	-1.0265E+00	-1.0316E+00	**-1.0316E+00**	-1.0313E+00	-1.0316E+00	-1.0316E+00	-1.0316E+00	-1.0316E+00	-1.0316E+00	-1.0316E+00	-1.0316E+00	-1.0301E+00
	Rank	12	8	**1**	10	6	4	3	5	2	7	9	11
	Average_RunTime	7.2900E-02	3.2000E-02	2.0220E-01	***2.9900E-02***	3.1100E-02	4.2330E-01	5.8300E-02	3.2504E+00	3.2849E+00	5.5840E-01	1.0330E-01	9.8000E-02
F17	Average	3.9890E-01	3.9790E-01	**3.9790E-01**	4.0030E-01	3.9790E-01	3.9790E-01	3.9790E-01	3.9790E-01	**3.9790E-01**	**3.9790E-01**	**3.9790E-01**	4.0210E-01
	StandDP	1.2000E-03	2.3686E-06	**0.0000E+00**	2.3000E-03	2.1641E-05	2.1306E-14	1.6519E-14	4.8071E-09	**0.0000E+00**	**0.0000E+00**	**0.0000E+00**	3.4000E-03
	Med	3.9860E-01	3.9790E-01	**3.9790E-01**	3.9910E-01	3.9790E-01	3.9790E-01	3.9790E-01	3.9790E-01	**3.9790E-01**	**3.9790E-01**	**3.9790E-01**	4.0070E-01
	BestVal	3.9790E-01	3.9790E-01	**3.9790E-01**	3.9800E-01	3.9790E-01	3.9790E-01	3.9790E-01	3.9790E-01	**3.9790E-01**	**3.9790E-01**	**3.9790E-01**	3.9800E-01
	WorstVal	4.0310E-01	3.9790E-01	**3.9790E-01**	4.0520E-01	3.9800E-01	3.9790E-01	3.9790E-01	3.9790E-01	**3.9790E-01**	**3.9790E-01**	**3.9790E-01**	4.0980E-01
	Rank	10	8	**1**	11	9	6	5	7	**1**	**1**	**1**	12
	Average_RunTime	4.6400E-02	2.1100E-02	1.4680E-01	1.9100E-02	***1.8900E-02***	3.1950E-01	3.8000E-02	2.3456E+00	2.3671E+00	4.0700E-01	6.2400E-02	6.9200E-02
F18	Average	3.0453E+00	3.0001E+00	**3.0000E+00**	3.0003E+00	3.0001E+00	3.0000E+00	3.0000E+00	3.0000E+00	3.0000E+00	3.0000E+00	3.0000E+00	3.0000E+00
	StandDP	4.9800E-02	7.6541E-05	**8.0799E-16**	8.6357E-04	1.1584E-04	1.2799E-12	9.3532E-13	1.3795E-13	1.4867E-15	1.0333E-15	2.1927E-15	2.8349E-05
	Med	3.0306E+00	3.0001E+00	**3.0000E+00**	3.0001E+00	3.0000E+00	3.0000E+00	3.0000E+00	3.0000E+00	3.0000E+00	3.0000E+00	3.0000E+00	3.0000E+00
	BestVal	3.0027E+00	3.0000E+00	**3.0000E+00**	3.0000E+00	3.0000E+00	3.0000E+00	3.0000E+00	3.0000E+00	3.0000E+00	3.0000E+00	3.0000E+00	3.0000E+00
	WorstVal	3.2316E+00	3.0003E+00	**3.0000E+00**	3.0048E+00	3.0004E+00	3.0000E+00	3.0000E+00	3.0000E+00	3.0000E+00	3.0000E+00	3.0000E+00	3.0001E+00
	Rank	12	9	**1**	11	10	7	6	5	3	2	4	8
	Average_RunTime	6.0600E-02	2.8100E-02	2.0050E-01	2.5500E-02	***2.4300E-02***	4.0740E-01	5.2800E-02	3.0056E+00	3.0320E+00	5.1920E-01	8.5000E-02	8.8500E-02
F19	Average	-3.8542E+00	-3.8613E+00	-3.8628E+00	-3.8523E+00	-3.8558E+00	-3.8628E+00	-3.8628E+00	-3.8613E+00	**-3.8628E+00**	-3.8628E+00	-3.8628E+00	-3.7024E+00
	StandDP	5.1000E-03	2.6000E-03	2.7101E-15	3.8000E-03	1.1600E-02	4.5177E-10	2.9935E-06	2.8000E-03	**2.6823E-15**	2.7101E-15	2.7101E-15	1.5840E-01
	Med	-3.8548E+00	-3.8626E+00	-3.8628E+00	-3.8540E+00	-3.8583E+00	-3.8628E+00	-3.8628E+00	-3.8627E+00	**-3.8628E+00**	-3.8628E+00	-3.8628E+00	-3.7605E+00
	BestVal	-3.8618E+00	-3.8628E+00	-3.8628E+00	-3.8596E+00	-3.8628E+00	-3.8628E+00	-3.8628E+00	-3.8628E+00	**-3.8628E+00**	-3.8628E+00	-3.8628E+00	-3.8511E+00
	WorstVal	-3.8409E+00	-3.8549E+00	-3.8628E+00	-3.8435E+00	-3.8013E+00	-3.8628E+00	-3.8628E+00	-3.8549E+00	**-3.8628E+00**	-3.8628E+00	-3.8628E+00	-3.2704E+00
	Rank	10	7	2	9	11	5	6	8	**1**	2	2	12
	Average_RunTime	5.7800E-02	2.8100E-02	1.6410E-01	2.6300E-02	***2.5800E-02***	4.3490E-01	4.5300E-02	3.3034E+00	3.3322E+00	4.3200E-01	8.8800E-02	9.2300E-02
F20	Average	-3.1761E+00	-3.2676E+00	**-3.3219E+00**	-2.9508E+00	-3.2366E+00	-3.2511E+00	-3.2133E+00	-3.2290E+00	-3.2247E+00	-3.2784E+00	-3.2427E+00	-2.3575E+00
	StandDP	9.3800E-02	7.4600E-02	**1.8394E-04**	2.3830E-01	9.6300E-02	6.3500E-02	6.4100E-02	8.9900E-02	5.0900E-02	5.8300E-02	5.7000E-02	3.7810E-01
	Med	-3.1945E+00	-3.3219E+00	**-3.3220E+00**	-3.0087E+00	-3.3037E+00	-3.2025E+00	-3.1973E+00	-3.1978E+00	-3.2031E+00	-3.3220E+00	-3.2031E+00	-2.3894E+00
	BestVal	-3.3067E+00	-3.3220E+00	**-3.3220E+00**	-3.1837E+00	-3.3216E+00	-3.3220E+00	-3.3220E+00	-3.3220E+00	-3.3220E+00	-3.3220E+00	-3.3220E+00	-3.0386E+00
	WorstVal	-2.8661E+00	-3.1363E+00	**-3.3213E+00**	-2.0347E+00	-3.0449E+00	-3.1712E+00	-3.1363E+00	-3.0797E+00	-3.1376E+00	-3.2031E+00	-3.2031E+00	-1.4768E+00
	Rank	9	7	**1**	11	10	5	6	8	2	4	3	12
	Average_RunTime	9.4500E-02	4.1200E-02	2.2090E-01	3.9100E-02	***3.5100E-02***	9.5170E-01	6.1200E-02	6.5955E+00	6.6371E+00	5.7120E-01	1.5740E-01	1.8750E-01
F21	Average	-9.8851E+00	-9.0631E+00	-9.9562E+00	-2.6550E+00	-8.0167E+00	-5.6911E+00	-7.0703E+00	-7.0340E+00	-6.4639E+00	-8.0990E+00	**-1.0153E+01**	-2.3721E+00
	StandDP	2.3820E-01	2.5116E+00	4.4520E-01	1.6928E+00	3.0866E+00	2.4387E+00	3.6338E+00	2.8650E+00	3.2077E+00	2.5253E+00	**7.0516E-15**	6.8360E-01
	Med	-9.9613E+00	-1.0147E+01	-1.0135E+01	-2.3449E+00	-1.0083E+01	-5.1008E+00	-1.0153E+01	-5.1008E+00	-5.1008E+00	-1.0135E+01	**-1.0153E+01**	-2.4370E+00
	BestVal	-1.0118E+01	-1.0152E+01	-1.0153E+01	-5.0110E+00	-1.0151E+01	-1.0153E+01	-1.0153E+01	-1.0153E+01	-1.0153E+01	-1.0153E+01	**-1.0153E+01**	-3.9775E+00
	WorstVal	-9.1315E+00	-2.6818E+00	-8.4269E+00	-4.9650E-01	-2.6254E+00	-2.6305E+00	-2.6305E+00	-2.6305E+00	-2.6305E+00	-5.0552E+00	**-1.0153E+01**	-1.1163E+00
	Rank	2	7	3	5	10	6	12	9	11	8	**1**	4
	Average_RunTime	7.8700E-02	3.6900E-02	1.7860E-01	3.3800E-02	***3.2300E-02***	5.6530E-01	5.2900E-02	3.2474E+00	3.2841E+00	4.6790E-01	1.1690E-01	1.2170E-01
