# Supplementary material for: A novel hybrid PSO based on levy flight and wavelet mutation for global optimization
Source: PLoS One. 2023 Jan 6;18(1):e0279572. doi: 10.1371/journal.pone.0279572 (PMC9821455; doi:10.1371/journal.pone.0279572)
Supplement: S8 Appendix — Numerical results of Wilcoxon’s rank sum test for PSOLFWM and other optimization algorithms in the optimization search process for benchmark test functions F1-F21 are given. (PDF) [file pone.0279572.s008.pdf]

Table 17. Comparison of PSOLFWM with other algorithms using Wilcoxon's rank sum test.

Function Name	Wilcoxon's rank-sum test	PSO [38]	SPSO [45]	HPSOM [46]	HPSOWM [28]	BBPSO [21]	PSOLF [41]	PSOSCALF [34]	PSOGWO [47]	GWO [3]	DE [48]	SCA [13]	WOA [4]	ALO [5]	SSA [6]	DA [12]	MFO [10]	BES [9]	CSA [17]	SSO [49]
F1	p-value	1.2118E-12	1.2118E-12	1.2118E-12	1.2118E-12	1.2118E-12	1.2118E-12	1.2118E-12	1.2118E-12	1.2118E-12	1.2118E-12	1.2118E-12	1.2118E-12	1.2118E-12	1.2118E-12	1.2118E-12	1.2118E-12	NaN	1.2118E-12	1.2118E-12
	h-value	1	1	1	1	1	1	1	1	1	1	1	1	1	1	1	1	0	1	1
	z-value	-7.1040E+00	-7.1040E+00	-7.1040E+00	-7.1040E+00	-7.1040E+00	-7.1040E+00	-7.1040E+00	-7.1040E+00	-7.1040E+00	-7.1040E+00	-7.1040E+00	-7.1040E+00	-7.1040E+00	-7.1040E+00	-7.1040E+00	-7.1040E+00	NaN	-7.1040E+00	-7.1040E+00
F2	p-value	3.0199E-11	3.0199E-11	3.0199E-11	3.0199E-11	3.0199E-11	3.0199E-11	3.0199E-11	3.0199E-11	3.0199E-11	3.0199E-11	3.0199E-11	3.0199E-11	3.0199E-11	3.0199E-11	3.0199E-11	3.0199E-11	1.2118E-12	3.0199E-11	3.0199E-11
	h-value	1	1	1	1	1	1	1	1	1	1	1	1	1	1	1	1	1	1	1
	z-value	-6.6456E+00	-6.6456E+00	-6.6456E+00	-6.6456E+00	-6.6456E+00	-6.6456E+00	-6.6456E+00	-6.6456E+00	-6.6456E+00	-6.6456E+00	-6.6456E+00	-6.6456E+00	-6.6456E+00	-6.6456E+00	-6.6456E+00	-6.6456E+00	7.1040E+00	-6.6456E+00	-6.6456E+00
F3	p-value	1.2118E-12	1.2118E-12	1.2118E-12	1.2118E-12	1.2118E-12	1.2118E-12	1.2118E-12	1.2118E-12	1.2118E-12	1.2118E-12	1.2118E-12	1.2118E-12	1.2118E-12	1.2118E-12	1.2118E-12	1.2118E-12	NaN	1.2118E-12	1.2118E-12
	h-value	1	1	1	1	1	1	1	1	1	1	1	1	1	1	1	1	0	1	1
	z-value	-7.1040E+00	-7.1040E+00	-7.1040E+00	-7.1040E+00	-7.1040E+00	-7.1040E+00	-7.1040E+00	-7.1040E+00	-7.1040E+00	-7.1040E+00	-7.1040E+00	-7.1040E+00	-7.1040E+00	-7.1040E+00	-7.1040E+00	-7.1040E+00	NaN	-7.1040E+00	-7.1040E+00
F4	p-value	3.0199E-11	3.0199E-11	3.0199E-11	3.0199E-11	3.0199E-11	3.0199E-11	3.0199E-11	3.0199E-11	3.0199E-11	3.0199E-11	3.0199E-11	3.0199E-11	3.0199E-11	3.0199E-11	3.0199E-11	3.0199E-11	1.2118E-12	3.0199E-11	3.0199E-11
	h-value	1	1	1	1	1	1	1	1	1	1	1	1	1	1	1	1	1	1	1
	z-value	-6.6456E+00	-6.6456E+00	-6.6456E+00	-6.6456E+00	-6.6456E+00	-6.6456E+00	-6.6456E+00	-6.6456E+00	-6.6456E+00	-6.6456E+00	-6.6456E+00	-6.6456E+00	-6.6456E+00	-6.6456E+00	-6.6456E+00	-6.6456E+00	7.1040E+00	-6.6456E+00	-6.6456E+00
F5	p-value	3.0199E-11	3.0199E-11	3.0199E-11	1.0666E-07	3.0199E-11	3.0199E-11	3.0199E-11	3.0199E-11	3.0199E-11	3.0199E-11	3.0199E-11	3.0199E-11	3.0199E-11	3.0199E-11	3.0199E-11	3.0199E-11	3.0199E-11	3.0199E-11	3.0199E-11
	h-value	1	1	1	1	1	1	1	1	1	1	1	1	1	1	1	1	1	1	1
	z-value	-6.6456E+00	-6.6456E+00	-6.6456E+00	-5.3150E+00	-6.6456E+00	-6.6456E+00	-6.6456E+00	-6.6456E+00	-6.6456E+00	-6.6456E+00	-6.6456E+00	-6.6456E+00	-6.6456E+00	-6.6456E+00	-6.6456E+00	-6.6456E+00	-6.6456E+00	-6.6456E+00	-6.6456E+00
F6	p-value	3.0199E-11	3.0199E-11	3.0199E-11	3.0199E-11	3.0199E-11	3.0199E-11	3.0199E-11	1.1077E-06	3.0199E-11	3.0199E-11	3.0199E-11	3.0199E-11	3.0199E-11	3.0199E-11	3.0199E-11	3.0199E-11	3.0199E-11	3.0199E-11	3.0199E-11
	h-value	1	1	1	1	1	1	1	1	1	1	1	1	1	1	1	1	1	1	1
	z-value	-6.6456E+00	-6.6456E+00	-6.6456E+00	6.6456E+00	-6.6456E+00	-6.6456E+00	-6.6456E+00	4.8715E+00	-6.6456E+00	-6.6456E+00	-6.6456E+00	-6.6456E+00	-6.6456E+00	-6.6456E+00	-6.6456E+00	-6.6456E+00	6.6456E+00	6.6456E+00	-6.6456E+00
F7	p-value	3.0199E-11	3.0199E-11	3.0199E-11	1.4110E-09	3.0199E-11	5.2010E-01	1.0937E-10	3.0199E-11	3.9648E-08	3.0199E-11	3.0199E-11	2.4327E-05	3.0199E-11	3.0199E-11	3.0199E-11	3.0199E-11	4.6159E-10	2.3768E-07	4.7445E-06
	h-value	1	1	1	1	1	0	1	1	1	1	1	1	1	1	1	1	1	1	1
	z-value	-6.6456E+00	-6.6456E+00	-6.6456E+00	-6.0542E+00	-6.6456E+00	-6.4310E-01	-6.4534E+00	-6.6456E+00	-5.4924E+00	-6.6456E+00	-6.6456E+00	-4.2210E+00	-6.6456E+00	-6.6456E+00	-6.6456E+00	-6.6456E+00	6.2316E+00	5.1672E+00	4.5758E+00
F8	p-value	4.9752E-11	3.0199E-11	3.0199E-11	4.5043E-11	4.0772E-11	3.0199E-11	4.9752E-11	3.0199E-11	2.8716E-10	5.5727E-10	3.0199E-11	3.5550E-01	2.8040E-11	8.8910E-10	1.9568E-10	6.5183E-09	1.2057E-10	5.1857E-07	1.3289E-10
	h-value	1	1	1	1	1	1	1	1	1	1	1	0	1	1	1	1	1	1	1
	z-value	-6.5717E+00	-6.6456E+00	-6.6456E+00	-6.5865E+00	6.6012E+00	-6.6456E+00	-6.5717E+00	-6.6456E+00	-6.3056E+00	-6.2021E+00	-6.6456E+00	-9.2400E-01	-6.6565E+00	-6.1281E+00	-6.3647E+00	-5.8029E+00	-6.4386E+00	-5.0193E+00	-6.4238E+00
F9	p-value	1.2118E-12	1.2118E-12	1.2118E-12	1.2118E-12	1.2118E-12	NaN	1.2118E-12	1.2118E-12	1.2118E-12	1.2118E-12	1.2118E-12	1.6080E-01	1.2118E-12	1.2118E-12	1.2118E-12	1.2118E-12	NaN	NaN	NaN
	h-value	1	1	1	1	1	0	1	1	1	1	1	0	1	1	1	1	0	0	0
	z-value	-7.1040E+00	-7.1040E+00	-7.1040E+00	-7.1040E+00	-7.1040E+00	NaN	-7.1040E+00	-7.1040E+00	-7.1040E+00	-7.1040E+00	-7.1040E+00	-1.4024E+00	-7.1040E+00	-7.1040E+00	-7.1040E+00	-7.1040E+00	NaN	NaN	NaN
Function Name	Wilcoxon's rank-sum test	PSO	SPSO	HPSOM	HPSOWM	BBPSO	PSOLF	PSOSCALF	PSOGWO	GWO	DE	SCA	WOA	ALO	SSA	DA	MFO	BES	CSA	SSO
F10	p-value	1.2118E-12	1.2118E-12	1.2118E-12	1.2118E-12	1.2118E-12	NaN	1.2118E-12	1.2118E-12	1.2118E-12	1.2118E-12	1.2118E-12	6.4721E-13	1.2118E-12	1.2118E-12	1.2118E-12	1.2118E-12	NaN	NaN	6.1783E-04
	h-value	1	1	1	1	1	0	1	1	1	1	1	1	1	1	1	1	0	0	1
	z-value	-7.1040E+00	-7.1040E+00	-7.1040E+00	-7.1040E+00	-7.1040E+00	NaN	-7.1040E+00	-7.1040E+00	-7.1040E+00	-7.1040E+00	-7.1040E+00	-7.1901E+00	-7.1040E+00	-7.1040E+00	-7.1040E+00	-7.1040E+00	NaN	NaN	-3.4237E+00
F11	p-value	1.2118E-12	1.2118E-12	1.2118E-12	1.2118E-12	NaN	NaN	1.2118E-12	1.2118E-12	1.2118E-12	1.2118E-12	1.2118E-12	1.6080E-01	1.2118E-12	1.2118E-12	1.2118E-12	1.2118E-12	NaN	NaN	NaN
	h-value	1	1	1	1	0	0	1	1	1	1	1	0	1	1	1	1	0	0	0
	z-value	-7.1040E+00	-7.1040E+00	-7.1040E+00	-7.1040E+00	NaN	NaN	-7.1040E+00	-7.1040E+00	-7.1040E+00	-7.1040E+00	-7.1040E+00	-1.4024E+00	-7.1040E+00	-7.1040E+00	-7.1040E+00	-7.1040E+00	NaN	NaN	NaN
F12	p-value	3.0199E-11	3.0199E-11	3.0199E-11	3.0199E-11	3.0199E-11	3.0199E-11	3.0199E-11	1.5292E-05	3.0199E-11	3.0199E-11	3.0199E-11	3.0199E-11	3.0199E-11	3.0199E-11	3.0199E-11	3.0199E-11	3.0199E-11	3.0199E-11	3.0199E-11
	h-value	1	1	1	1	1	1	1	1	1	1	1	1	1	1	1	1	1	1	1
	z-value	-6.6456E+00	-6.6456E+00	-6.6456E+00	6.6456E+00	-6.6456E+00	-6.6456E+00	-6.6456E+00	-4.3244E+00	-6.6456E+00	-6.6456E+00	-6.6456E+00	-6.6456E+00	-6.6456E+00	-6.6456E+00	-6.6456E+00	-6.6456E+00	6.6456E+00	6.6456E+00	-6.6456E+00
F13	p-value	3.0199E-11	3.0199E-11	3.0199E-11	8.1014E-10	3.0199E-11	3.0199E-11	3.0199E-11	6.6955E-11	3.0199E-11	3.0199E-11	3.0199E-11	3.0199E-11	3.0199E-11	3.0199E-11	3.0199E-11	3.0199E-11	2.9878E-11	1.4130E-01	3.0199E-11
	h-value	1	1	1	1	1	1	1	1	1	1	1	1	1	1	1	1	1	0	1
	z-value	-6.6456E+00	-6.6456E+00	-6.6456E+00	6.1429E+00	-6.6456E+00	-6.6456E+00	-6.6456E+00	-6.5273E+00	-6.6456E+00	-6.6456E+00	-6.6456E+00	-6.6456E+00	-6.6456E+00	-6.6456E+00	-6.6456E+00	-6.6456E+00	-6.6472E+00	-1.4711E+00	-6.6456E+00
F14	p-value	2.1959E-07	5.4998E-10	5.3000E-03	1.1070E-06	3.4030E-01	2.1265E-04	3.0811E-08	1.7836E-04	9.3300E-02	1.2118E-12	3.0199E-11	1.6300E-02	2.6600E-02	1.9000E-03	3.2530E-01	6.7000E-03	9.6200E-02	2.6700E-02	3.6897E-11
	h-value	1	1	1	1	0	1	1	1	0	1	1	1	1	1	0	1	0	1	1
	z-value	5.1819E+00	-6.2041E+00	-2.7869E+00	-4.8716E+00	-9.5360E-01	-3.7035E+00	-5.5368E+00	3.7479E+00	-1.6780E+00	7.1040E+00	-6.6456E+00	-2.4025E+00	-2.2166E+00	3.1122E+00	-9.8370E-01	2.7129E+00	1.6635E+00	-2.2157E+00	-6.6160E+00
F15	p-value	3.4742E-10	1.1737E-09	5.4941E-11	5.3000E-03	3.0199E-11	3.8307E-05	1.1058E-04	8.2400E-02	3.8000E-03	4.9752E-11	5.4941E-11	9.5332E-07	1.6132E-10	1.0105E-08	3.0161E-11	8.1014E-10	8.0505E-08	4.3106E-08	2.9200E-02
	h-value	1	1	1	1	1	1	1	0	1	1	1	1	1	1	1	1	1	1	1
	z-value	-6.2760E+00	-6.0838E+00	-6.5569E+00	2.7869E+00	-6.6456E+00	-4.1175E+00	-3.8661E+00	-1.7372E+00	-2.8904E+00	-6.5717E+00	-6.5569E+00	-4.9010E+00	-6.3943E+00	-5.7290E+00	-6.6458E+00	-6.1429E+00	5.3660E+00	-5.4776E+00	-2.1807E+00
F16	p-value	3.0199E-11	1.0149E-11	3.3384E-11	3.0199E-11	1.4733E-07	3.0199E-11	1.2023E-08	6.6955E-11	3.0199E-11	5.1436E-12	1.6132E-10	3.0199E-11	3.0199E-11	3.0161E-11	1.6536E-11	3.1507E-12	1.7203E-12	2.3657E-12	5.9706E-05
	h-value	1	1	1	1	1	1	1	1	1	1	1	1	1	1	1	1	1	1	1
	z-value	6.6456E+00	6.8044E+00	6.6308E+00	6.6456E+00	-5.2559E+00	6.6456E+00	5.6994E+00	6.5273E+00	6.6456E+00	6.9016E+00	6.3943E+00	6.6456E+00	6.6456E+00	6.6458E+00	6.7337E+00	6.9708E+00	7.0555E+00	7.0110E+00	4.0140E+00
F17	p-value	3.0199E-11	1.2118E-12	3.3384E-11	3.0199E-11	8.8910E-10	1.2870E-09	3.0300E-02	6.0658E-11	3.0199E-11	1.2118E-12	6.1000E-03	1.7769E-10	2.9841E-11	2.9897E-11	4.1110E-12	1.2118E-12	1.2118E-12	1.2118E-12	1.0907E-05
	h-value	1	1	1	1	1	1	1	1	1	1	1	1	1	1	1	1	1	1	1
	z-value	6.6456E+00	7.1040E+00	6.6308E+00	6.6456E+00	-6.1281E+00	6.0690E+00	2.1659E+00	6.5421E+00	6.6456E+00	7.1040E+00	-2.7425E+00	6.3795E+00	6.6474E+00	6.6471E+00	6.9333E+00	7.1040E+00	7.1040E+00	7.1040E+00	-4.3984E+00
F18	p-value	3.0199E-11	2.2391E-11	3.0199E-11	3.0199E-11	1.5292E-05	3.0199E-11	1.6132E-10	3.0199E-11	3.0199E-11	1.4239E-11	3.6897E-11	3.0199E-11	3.0180E-11	3.0199E-11	2.9766E-11	2.5399E-11	6.4605E-12	2.7081E-11	3.0199E-11
	h-value	1	1	1	1	1	1	1	1	1	1	1	1	1	1	1	1	1	1	1
	z-value	6.6456E+00	6.6895E+00	6.6456E+00	6.6456E+00	-4.3244E+00	6.6456E+00	6.3943E+00	6.6456E+00	6.6456E+00	6.7555E+00	6.6160E+00	6.6456E+00	6.6457E+00	6.6456E+00	6.6477E+00	6.6710E+00	6.8691E+00	6.6616E+00	6.6456E+00
F19	p-value	1.0000E-03	9.1747E-12	2.8716E-10	3.0199E-11	3.1500E-02	9.2603E-09	8.7000E-03	3.0199E-11	1.3111E-08	1.2118E-12	4.5100E-02	5.6000E-03	3.0199E-11	3.0199E-11	1.8492E-08	2.3638E-12	1.2118E-12	1.2118E-12	2.1544E-10
	h-value	1	1	1	1	1	1	1	1	1	1	1	1	1	1	1	1	1	1	1
	z-value	3.2895E+00	6.8189E+00	6.3056E+00	6.6456E+00	-2.1511E+00	5.7437E+00	2.6242E+00	6.6456E+00	5.6846E+00	7.1040E+00	-2.0033E+00	2.7721E+00	6.6456E+00	6.6456E+00	5.6256E+00	7.0111E+00	7.1040E+00	7.1040E+00	-6.3499E+00
F20	p-value	1.6813E-04	8.5000E-02	4.2040E-01	6.9520E-01	6.5277E-08	8.8829E-06	7.2830E-01	6.1500E-02	6.7650E-05	3.0199E-11	4.6856E-08	8.0000E-03	1.7000E-03	2.3980E-01	4.2100E-02	5.3700E-02	4.0620E-06	4.2000E-03	4.0772E-11
	h-value	1	0	0	0	1	1	0	0	1	1	1	1	1	0	1	0	1	1	1
	z-value	-3.7626E+00	1.7224E+00	8.0580E-01	3.9180E-01	-5.4037E+00	4.4427E+00	-3.4740E-01	1.8702E+00	3.9844E+00	6.6456E+00	-5.4628E+00	2.6538E+00	3.1417E+00	1.1754E+00	2.0329E+00	1.9294E+00	4.6082E+00	2.8617E+00	-6.6012E+00
F21	p-value	2.5000E-03	1.9000E-03	8.7660E-01	1.0000E+00	3.0199E-11	3.1830E-01	2.0058E-04	6.0459E-07	9.5139E-06	8.6634E-05	3.0199E-11	1.5370E-01	6.7650E-05	3.7900E-01	3.7900E-01	1.8460E-01	4.1030E-01	2.3657E-12	3.0199E-11
	h-value	1	1	0	0	1	0	1	1	1	1	1	0	1	0	0	0	0	1	1
	z-value	3.0234E+00	-3.0990E+00	1.5520E-01	0.0000E+00	6.6456E+00	9.9790E-01	-3.7183E+00	4.9897E+00	4.4279E+00	3.9253E+00	-6.6456E+00	1.4267E+00	-3.9844E+00	8.7970E-01	-8.7970E-01	-1.3268E+00	8.2340E-01	7.0110E+00	-6.6456E+00
